# Sensory, psychological, and metabolic dysfunction in HIV-associated peripheral neuropathy: A cross-sectional deep profiling study

**DOI:** 10.1016/j.pain.2014.06.014

**Published:** 2014-09

**Authors:** Tudor J.C. Phillips, Matthew Brown, Juan D. Ramirez, James Perkins, Yohannes W. Woldeamanuel, Amanda C. de C. Williams, Christine Orengo, David L.H. Bennett, Istvan Bodi, Sarah Cox, Christoph Maier, Elena K. Krumova, Andrew S.C. Rice

**Affiliations:** a Pain Research Group, Department of Surgery and Cancer, Faculty of Medicine, Imperial College London, Chelsea and Westminster Hospital Campus, London, UK; b Nuffield Department of Clinical Neurosciences, Oxford University, UK; c Department of Bioinformatics, University College London, UK; d Department of Neurology, Addis Ababa University School of Medicine, Addis Ababa, Ethiopia; e Research Department of Clinical, Educational, and Health Psychology, University College London, UK; f Department of Neuropathology, Kings College London, UK; g Pain Medicine, Chelsea and Westminster Hospital NHS Foundation Trust, London, UK; h Department of Pain Management, BG University Hospital, Bochum, Germany; i Department of Neurology, BG University Hospital, Bochum, Germany

**Keywords:** Anxiety, Catastrophizing, Depression, Diagnosis, HIV-SN, IENFD, Pain, Phenotyping, QST, Quality of life, Triglycerides

## Abstract

HIV-associated sensory neuropathy (HIV-SN) is a frequent complication of HIV infection and a major source of morbidity. A cross-sectional deep profiling study examining HIV-SN was conducted in people living with HIV in a high resource setting using a battery of measures which included the following: parameters of pain and sensory symptoms (7 day pain diary, Neuropathic Pain Symptom Inventory [NPSI] and Brief Pain Inventory [BPI]), sensory innervation (structured neurological examination, quantitative sensory testing [QST] and intraepidermal nerve fibre density [IENFD]), psychological state (Pain Anxiety Symptoms Scale-20 [PASS-20], Depression Anxiety and Positive Outlook Scale [DAPOS], and Pain Catastrophizing Scale [PCS], insomnia (Insomnia Severity Index [ISI]), and quality of life (Short Form (36) Health Survey [SF-36]). The diagnostic utility of the Brief Peripheral Neuropathy Screen (BPNS), Utah Early Neuropathy Scale (UENS), and Toronto Clinical Scoring System (TCSS) were evaluated. Thirty-six healthy volunteers and 66 HIV infected participants were recruited. A novel triumvirate case definition for HIV-SN was used that required 2 out of 3 of the following: 2 or more abnormal QST findings, reduced IENFD, and signs of a peripheral neuropathy on a structured neurological examination. Of those with HIV, 42% fulfilled the case definition for HIV-SN (*n* = 28), of whom 75% (*n* = 21) reported pain. The most frequent QST abnormalities in HIV-SN were loss of function in mechanical and vibration detection. Structured clinical examination was superior to QST or IENFD in HIV-SN diagnosis. HIV-SN participants had higher plasma triglyceride, concentrations depression, anxiety and catastrophizing scores, and prevalence of insomnia than HIV participants without HIV-SN.

## Summary

Study participants with HIV-associated sensory polyneuropathy (HIV-SN) had higher plasma triglyceride concentrations, depression, anxiety, catastrophizing scores, and prevalence of insomnia than HIV participants without HIV-SN.

The current global prevalence of HIV is about 34 million [29]. HIV-associated sensory polyneuropathy (HIV-SN) is a distal symmetrical, predominantly sensory, polyneuropathy characterised by a “dying back” pattern of axonal degeneration. It is a frequent and often painful complication of HIV infection, and its treatment [31], [12], [13] with both viral–immune interactions and toxicity of the nucleoside reverse transcriptase inhibitor (NRTI) class of antiretroviral drugs (eg, stavudine [D4T]) potentially contributes to its pathogenesis [28], [30], [39], [68], [69]. The introduction of combination antiretroviral treatment (cART) in the mid-1990s transformed HIV infection from a high-mortality condition into a chronic disease, with the life expectancy of newly diagnosed individuals now approaching that of the general population [64], [45]. Notwithstanding this, HIV-SN continues to be one of the most prevalent morbidities experienced by people living with HIV in both high-resource and low-resource settings. Consequently, elucidating the nature of and developing strategies for the prevention and management of HIV-SN has become increasingly important.

HIV-SN affects between 27% and 57% of ambulatory HIV-infected individuals; of those with HIV-SN, 38% to 90% experience pain [4], [22], [31], [40], [49], [62]. Neuropathic pain associated with HIV-SN is often debilitating, adversely affecting quality of life [32]; it is also clinically difficult to treat [50]. Consequently, painful HIV-SN represents one of the largest causes of pain morbidity worldwide.

Established risk factors associated with the development of HIV-SN include advancing age, height, and exposure to the neurotoxic dNRTI (nucleoside reverse transcriptase inhibitor) class of antiretroviral agents [30], [31]. The dNRTI drugs are zalcitabine (ddC), stavudine (d4T), and didanosine (ddI). Crucially, despite the phasing out of neurotoxic dNRTI drugs, especially in well-resourced countries, the prevalence of HIV-SN has consistently remained at around 40%, even in people who have no history of exposure to dNRTIs, suggesting that drug-induced neurotoxicity may not be a major aetiological factor for HIV-SN in the cART era [12], [22], [62]. More recent cART-era population and gene association studies have identified additional patient-related risk factors such as ethnicity and elevated plasma triglycerides [4], as well as the association of genes involved in the peripheral inflammatory response and those affecting mitochondrial function [31]. Although there have been several epidemiological studies documenting the prevalence and risk factors for HIV-SN in the current cART era [12], [22], [62], profiling studies directed at describing in detail the HIV-SN phenotype are lacking. Specifically, factors which are crucial to understanding the nature of HIV-SN, such as the relationships between sensory nerve dysfunction, symptomatology, clinical signs, patient quality of life, psychological comorbidity, and sleep disturbance, are required. Although some early studies did describe aspects of sensory dysfunction in the type of patients appropriate to that era (ie, severely immunosuppressed patients, usually with AIDS), to our knowledge, there have been no studies detailing the HIV-SN sensory profile in the cART era, where the disease has a very different clinical presentation and natural history [8], [41].

We therefore conducted a detailed deep-profile study of a cohort of HIV-infected patients with and without HIV-SN, using a battery of techniques directed at understanding the nature and pattern of sensory nerve dysfunction in HIV-SN and its relationship to symptomatology, psychological morbidity, and circadian rhythm disruption.

## 2 Material and methods

### 2.1 Study design and patients

The Pain in Neuropathy Study—HIV (PINS-HIV) was an observational single cohort cross-sectional study conducted at Chelsea and Westminster Hospital in London. The study protocol was assessed and approved by a local ethics committee (Riverside Research Ethics Committee; NRES 09/H0706/24). Subjects participated in the study after giving written informed consent.

Participants were recruited between July 7, 2009, and January 25, 2011, from ambulatory HIV-infected patients attending the St Stephen’s Centre, Chelsea and Westminster Hospital. In order to reflect the population treated at one of Europe’s major HIV centres, the inclusion criteria were as inclusive as possible. Eligible persons were all HIV-infected adults (⩾18 years of age), irrespective of concurrent or previous antiretroviral (ARV) therapy, or the presence of symptoms of a peripheral neuropathy. Exclusion criteria included pregnancy, coincident major psychiatric disorders (DSM-IV), poor or no English language skills, and ⩾4 of 10 numerical rating scale (NRS) pain at recruitment from a cause other than a peripheral neuropathy (to prevent potential confounding influence on pain as well as psychological and quality-of-life patient-reported outcomes), patients with documented central nervous system lesions, or subjects with insufficient mental capacity for obtaining informed consent or to complete questionnaires. Skin biopsies were not conducted on anticoagulated participants or those who had other contraindication to skin biopsy.

The study design consisted of a single clinical assessment appointment, at the end of which participants were given a questionnaire pack to complete and return to the study centre by mail.

During the clinical assessment appointment, participants had detailed medical and drug histories taken by a study investigator, who recorded the following: gender, age, ethnicity, medical history, date of HIV diagnosis, presence of a family history of neuropathy, presence of other potential causes of neuropathy (hypothyroidism, diabetes, alcohol abuse, vitamin B_12_ deficiency, and isoniazid and chemotherapy drug exposure); smoking and alcohol consumption were assessed using UK Department of Health methodology [27].

Basic clinical parameters were then measured for each participant (weight, height, and lying/standing blood pressures). Participants then underwent a structured neurological examination (SNE), a detailed quantitative sensory testing (QST) assessment, and skin biopsy, as described below. Each participant had 30 mL of blood drawn and stored for future genotype studies.

After the clinical assessment, the study investigator collected further drug, laboratory, and clinical investigation data from the clinical records, where available, including detailed ARV drug histories; nerve conduction study data; and the most recent routine haematological and biochemical parameters, including HIV virus load, CD4^+^ counts, plasma electrolytes, liver function enzymes, vitamin B_12_, thyroid function, blood glucose, plasma lipid profiles, and hepatitis B, hepatitis C, and syphilis serology (Supplementary Document 1).

### 2.2 Quantitative sensory testing (QST)

Sensory profiles were measured using the German Research Network on Neuropathic Pain (DFNS) QST protocol. Measurements were performed bilaterally in the S1 dermatome (dorsum of the feet). The DFNS has developed and validated a comprehensive QST battery which uses standardised equipment, paradigms, and verbal instructions as described [57], [58]. This method has been used in multiple investigations of different neuropathic pain conditions to phenotypically characterise patterns of sensory nerve dysfunction [25], [33], [38].

The DFNS QST protocol assesses the functional characteristics of both small and large afferent fibres by recording responses to 13 thermal and mechanical stimuli. These are described elsewhere in detail [57], [58]. In brief, the DFNS QST battery tests the following modalities in the order: cold detection threshold (CDT), warm detection threshold (WDT), thermal sensory limen (TSL), the presence of paradoxical heat sensations (PHS), cold pain threshold (CPT), heat pain threshold (HPT), mechanical detection threshold (MDT), mechanical pain threshold (MPT), a stimulus response function for pinprick sensitivity (mechanical pain sensitivity [MPS]), allodynia (dynamic mechanical allodynia [DMA]), wind-up ratio (WUR), vibration detection threshold (VDT), and blunt pressure pain threshold (PPT).

The investigators (TP and MB) underwent a formal course of instruction in conducting the DFNS QST protocol at Mannheim University and BG University hospital, Bochum, respectively, using healthy volunteers. On return to the UK investigation centre, for quality control purposes, each investigator was required to produce QST data sets for 18 healthy volunteer controls which were age and sex matched to DFNS requirements to ensure an equal number of male and female participants. Inclusion and exclusion criteria are detailed in Supplementary Document 2.

The healthy volunteer control data were subsequently analysed and critiqued by the DFNS to provide quality assurances for the study centre and QST investigators. Additionally, normative data for suprathreshold heat testing were collected from each healthy control subject after the completion of the DFNS QST protocol (Supplementary Document 2).

*QST equipment.* DFNS specification compliant QST equipment was used in the study as described in Supplementary Document 2.

*QST data analysis.* QST data entry was into an Excel-based (Excel 2007; Microsoft) data analysis system (Equista) provided by the DFNS. This system allowed entry of basic patient demographics and QST data. Equista performed z-score transformations of raw QST data values by comparing against normative reference data published by the DFNS (*n* = 180 subjects, bilateral assessment of 560 test areas) [37]. The DFNS normative reference data include age, sex, and anatomical test site matched. The mathematical transformation of QST data to z-scores has been described elsewhere in detail [57].

We utilized the DFNS coding system [38] to examine combinations of sensory dysfunction in HIV-SN. Accordingly, a value for a QST parameter within the normal DFNS reference range was designated 0; the presence of thermal hypoesthesia (ie, loss of WDT or CDT) was designated as L1; and the presence of hypoesthesia to mechanical modalities (ie, loss of MDT or VDT) was designated L2. Gain of sensory function to thermal modalities was designated G1 and gain of sensory function to mechanical modalities as G2. When both thermal and mechanical abnormalities were present, they were designated as L3 and G3, respectively.

### 2.3 Heat suprathreshold nociceptive testing

We included a stimulus response function for suprathreshold thermal stimuli. After completion of the DFNS QST protocol, participants were exposed to 14 suprathreshold heat stimuli in a temperature range of 44°C to 52°C at 2°C intervals (Supplementary Document 2).

As with the DFNS QST protocol, suprathreshold thermal stimuli were generated using an MSA100 thermal stimulator (Somedic AB), which uses a fluid-cooled Peltier element thermode measuring 25 × 50 mm.

### 2.4 Structured neurological examination

A comprehensive structured upper and lower limb neurological examination was devised to detect clinical signs of a peripheral neuropathy. The examination was performed on each patient and included assessment of light touch and pinprick sensation, joint position proprioception, vibration perception, deep-tendon reflexes, muscle wasting, and motor power (Supplementary Document 3). An abnormal result was taken as 2 or more symmetrical signs in the hands or feet consistent with a peripheral neuropathy.

Sympathetic nervous system function was examined by testing for the presence of orthostatic hypotension, as assessed by measuring lying and standing blood pressure in accordance with established protocols [16]. Lying and standing blood pressures were each measured in triplicate using a noninvasive blood pressure measuring system (Patient Transport Monitor HP M1275A, Hewlett-Packard). Lying blood pressures were measured first, after which the subject was asked to stand for 3 min so standing blood pressure could be measured. Orthostatic hypotension was determined to be present in subjects in whom either at least a 20 mm Hg reduction in systolic or a 10 mm Hg reduction in diastolic blood pressure was observed.

### 2.5 Intraepidermal nerve fibre density testing

The determination of IENFD in skin biopsy samples is a validated and sensitive diagnostic tool for the assessment of small fibre neuropathies, including HIV-SN [26], [35], [36], [54], [55].

Punch biopsies of skin were performed immediately after the completion of QST. Biopsy samples were taken in accordance with the consensus document produced by the European Federation of Neurological Societies/Peripheral Nerve Society Guideline [35], [36] on the utilisation of skin biopsy samples in the diagnosis of peripheral neuropathies. After local infiltration of skin with 1% lidocaine and under sterile conditions, skin biopsies were performed from a site 10 cm proximal to the lateral malleolus with a disposable 3 mm punch biopsy circular blade (Stiefel Laboratories Inc, GSK Plc).

The freshly collected biopsy samples were fixed for 12 to 24 h in 2% paraformaldehyde/lysine/periodate fixative at 4°C and rinsed with 0.08% Sorensen phosphate buffer. Samples underwent cryoprotection with 15% and then 30% sucrose solutions for 24 h each and were then embedded in OCT (Fisher Scientific UK Ltd), snap-frozen by submersion in liquid nitrogen, and stored at −20°C. Each biopsy was cut into 50 μm sections with a sliding microtome. Immunohistochemistry for PGP 9.5 (Ultraclone Ltd; dilution 1:15,000) was performed on sections using the immunoperoxidase method. Sections from each patient were processed in 2 separate staining runs to eliminate the risk of tissue loss and to ensure staining quality. All sections were allocated an individual code, and 3 sections per subject were selected randomly to undergo IENFD analysis using random sequences generated by an online tool (http://www.random.org/).

IENFD was assessed using a double bright-field microscope at 40× magnification using established counting rules [35], [36]. The 2 IENFD assessors (MB and JR) underwent instruction and technical validation in IENFD assessment at a clinical diagnostic laboratory overseen by one of the authors (IB). The study biopsy samples were coded so that the assessors were unaware of the participant’s neuropathy status. For the study materials, each assessor independently determined IENFD values on 3 biopsy sections for each participant; the resulting mean value was used. Interobserver Pearson’s product-moment correlation coefficients were determined for all the samples between the 2 microscopists; in addition, intraobserver reliability scores were also determined for 20 of these study sample selected randomly (http://www.random.org/).

In accordance with previously published data, IENFD values below 7.63 fibre/mm were considered to be abnormal [3]. This value has been shown to be associated with a specificity of 90% and sensitivity of 82.8% for the diagnosis of small fibre neuropathies [20].

### 2.6 Neuropathy screening tools

*Brief Peripheral Neuropathy Screen (BPNS).* The BPNS tool was developed for use by nonspecialist medical personnel to detect HIV-SN and has been used in several studies [14], [21], [60]. The BPNS consists of a set of questions relating to patient-reported symptoms of a peripheral neuropathy and a brief examination of the distal lower limb vibration perception and deep tendon reflexes.

The subjective component of the BPNS asks patients to report the presence of pain, aching, burning in the feet and/or legs and to report the presence of ‘pins and needles’ and numbness in feet and/or legs. Symptom severity is scored on an 11-point scale, with 0 = absent and 10 = severe.

The lower limb examination consists of vibration perception evaluation using a 128 Hz tuning fork maximally struck and placed on the top of the distal interphalangeal joint on 1 great toe. The time for a patient to lose vibration perception is graded as follows: grade 0, >10 s; grade 1, felt for 6 to 10 s; grade 2, felt for 5 s or less; and grade 3, no perception of vibration. Ankle reflexes were assessed relative to knee reflexes and graded as follows: grade 0, absent; grade 1, hypoactive; grade 2, normal; grade 4, clonus.

As originally described, the BPNS is not proscriptive on the definition of peripheral neuropathy used, and several studies have used different definitions for diagnosing HIV-SN. We used the method described by Cherry et al. [14], which has also been used in a number of recent studies [1], [10], [11], [72] and which requires both symptoms and signs. Subjects are considered to have HIV-SN if they have at least 1 symptom *and* either reduced or absent vibration sensation *or* ankle reflexes*.*

*Toronto Clinical Scoring System (TCSS).* The TCSS was developed as a screening tool for diabetic peripheral neuropathy [48]. Subsequent studies validated its utility in reflecting diabetic neuropathy severity, its correlation with clinical electrophysiological measures, and microscopic morphological changes in peripheral nerve biopsy samples [9]. The TCSS uses a simplified neurological examination assessing peripheral sensory perception, deep tendon reflexes, and the presence of neuropathy symptoms.

Sensory testing is performed on the first toe for the following: pinprick sensation using a Neurotip pin (Owen Mumford); temperature discrimination (warm- and cool-water-filled test tubes); light touch with a 10 g monofilament; and vibration with a maximally struck 128 Hz tuning fork, performed at the first toe. Responses were rated as normal or abnormal and were assigned 0 or 1 point, respectively. Scores range from 0 to a maximum of 19.

Deep tendon reflexes scores are graded for each side as loss, 2; reduced, 1; and normal, 0. The presence of each following neuropathy symptom scores 1 point: pain, numbness, tingling and weakness in the feet; the presence of similar upper limb symptoms; and the presence of unsteadiness on ambulation.

*Utah Early Neuropathy Scale (UENS).* The UENS is a physical examination scale developed specifically for the detection of early sensory-predominant diabetic polyneuropathy [61]. The emphasis of UENS is on the severity and spatial distribution of pin-evoked sharp sensation loss in the lower limb. The scale was developed and validated in a population with early diabetic peripheral neuropathy.

The UENS assesses sharp sensation in the lower leg relative to an unaffected portion of skin. A Neurotip pin is applied to the dorsum of the first toe and, working centripetally in 2 cm increments, the subject is asked at each application if they feel “any sharpness,” and if they do, whether it is “as sharp as they would expect.” This is performed on both lower limbs. The lower limbs are divided into 6 regions for testing; 2 points are scored for each region in which the subject fails to feel any sharpness. This is conducted and scored on both lower limbs. One additional point is scored for each additional region in which the pin feels less sharp than expected. Vibration is tested using a 128 Hz tuning fork maximally struck and applied to the dorsum of the great toe at the interphalangeal joint.

The UENS has also been correlated with data from electrophysiological and QST testing [61]. The UENS has recently been used in a small HIV-SN population, where it was shown to correlate with autonomic dysfunction measures (quantitative sudomotor axon reflex testing) and pain severity, but it was not validated for the diagnosis of HIV-SN [6].

### 2.7 Pain symptomatology, sleep disturbance, quality-of-life, and psychological comorbidity measures

Participants were given a questionnaire pack to complete and return to the investigation centre after their clinical assessment appointment. For the purpose of the questionnaires, participants were asked to consider any pain they were experiencing or the last time they had experienced pain. The body site was not prespecified.

*Seven-day pain diary.* Patients were asked to keep a pain intensity diary for 7 days, recording pain at 8 am and 8 pm daily on an 11 point scale, with 0 being no pain and 10 the worst pain imaginable.

*Neuropathic Pain Symptom Inventory (NPSI).* The NPSI is a validated self-administered questionnaire designed to evaluate neuropathic pain symptomatology [7]. It evaluates the presence and severity of 10 different neuropathic pain descriptors, each on an 11 point scale where 0 indicates no symptoms and 10 indicates maximal symptoms experienced. The NPSI also includes 2 temporal items assessing the duration of spontaneous ongoing pain, and the number of pain attacks on 5-point categorical scales; these temporal scores were not used in the final analysis. NPSI descriptor responses were divided into mild (0–3), moderate (4–6), and severe (7–10), and the proportion of participants experiencing each was determined [24].

Spearman correlation analysis was performed to assess correlations between QST and NPSI domains; responses were considered correlated if *r* > 0.3 and *P* < .0001.

*Brief Pain Inventory (BPI) 7-item pain interference subscale.* Pain-related interference in activities of daily living was assessed using the 7-item Pain Interference scale of the BPI [15]. The scale assesses pain interference within 7 domains: general activity, walking, work, relationships, mood, life enjoyment, and sleep. Participants score these on a 11 point scale ranging from 0 (does not interfere) to 10 (completely interferes). The composite score was calculated as the sum of the 7 interference items. Validity for the BPI comes from several studies of cancer pain and other diseases of pain. The BPI also demonstrates good test–retest item correlations over short time intervals [15], [19].

*SF-36 instrument.* Short Form 36 of the MOS Outcomes Study (SF-36) is an established instrument used for the assessment of health-related quality of life [70]. SF-36 responses were scored in the 8 domains of physical functioning: role–physical, bodily pain, general health, vitality, social functioning, role–emotional, and mental health. SF-36 scores range from 0 to 100, representing extreme dysfunction/symptom severity to optimal function, respectively. Published reliability statistics for the SF-36 for internal consistency and test–retesting have exceeded the minimum standard of 0.70 in group comparisons in more than 25 studies [63]. Relative to the longer measures that the SF-36 was constructed to reproduce, SF-36 scales have been shown to achieve about 80% to 90% of their empirical validity in studies involving physical and mental health criteria [44].

*Pain Anxiety Symptom Scale 20 (PASS-20).* Pain-related anxiety was assessed with the PASS-20 [42], which is a shortened version of the PASS [43]. PASS-20 has 20 questions assessing 4 facets of pain-related anxiety: fearfulness of pain, cognitive anxiety, escape/avoidance, and psychological anxiety. Each subscale score ranges from 0 (no interference) to 25 (maximum interference). Additionally, summation of the subscales provides a general measure of pain-related anxiety. The original long version of the PASS has been shown to correlate with measures of pain-related anxiety and fear [42]. The shortened PASS-20 scale has been shown to have a similar performance to the original PASS across the shortened and corresponding matching original scales (mean *r* = 0.95); the scale also has high internal consistency through item intercorrelations (mean α = 0.81).

*Amended Depression Anxiety Positive-Outlook instrument (DAPOS).* We used the depression and anxiety subscales of the DAPOS instrument [52]. This instrument was designed to measure mood specifically in pain populations and consists of 3 subscales. The subscale for depression (DAPOS-D) contains 5 items with scores ranging from 5 to 25, indicating normal mood to severe depression. The subscale for anxiety (DAPOS-A) contains 3 items, which ranges from 3 to 15 (no anxiety to maximal anxiety). The positive outlook component was omitted. The DAPOS has shown good internal consistency, with α ranging from 0.74 to 0.90. The DAPOS has also been shown to have good construct validity in comparison to several established measures (SF-36, Pain Catastrophizing Scale, Hospital Anxiety and Depression Scale (HADS) and Zung depression scale) [51], [52].

*Pain Catastrophizing Scale (PCS).* The PCS assesses the cognitive process by which pain is appraised in terms of threat and negative consequences [47]. It consists of 13 descriptions of thoughts and feelings related to pain. Respondents are asked to indicate the degree to which they experience these on a 5-point rating scale from 0 (not at all) to 4 (always). A high total score indicates a high level of pain catastrophizing. The instrument comprises 3 dimensions: rumination, magnification, and helplessness. Rumination refers to the patients’ preoccupation with pain; magnification expresses the exaggerated cognitions of pain as a threat; and hopelessness is patients’ feelings that they are unable to influence their pain. The PCS has been shown to have a high internal consistency and validity, with a high test–retest correlation (*r* = 0.75) over a 6-week interval in the same individual [47].

*Insomnia Severity Index (ISI).* To assess the prevalence of sleep dysfunction, we used the ISI [5]. The ISI was specifically designed to assess insomnia and is a brief self-report instrument, which measures a patient’s perception, subjective symptoms, and consequences of their insomnia. Its content corresponds, in part, to the diagnostic criteria of insomnia. The ISI is composed of 7 items assessing the severity of sleep onset and sleep maintenance difficulties, satisfaction with sleep patterns, interference with daily functioning, noticeability of impairment due to sleep dysfunction, and the degree of distress experienced by the patient.

### 2.8 Case definition of HIV-SN

Different case definitions have been proposed and used in earlier investigations of HIV-SN [8], [22], [62]. Most previous HIV-SN studies have assessed a combination of symptoms and basic clinical signs to detect peripheral neuropathies. The absence of a gold-standard definition for HIV-SN and for other small fibre neuropathies has been identified as a factor hindering progress in understanding HIV-SN [23] and small fibre neuropathy (SFN) pathophysiologies per se [20]. In this study, we propose a definition of HIV-SN using the criterion of 2 or more out of the following 3 items: clinical signs of distal sensory neuropathy elicited using the SNE, 2 or more abnormal QST findings using the full 13 parameters of the DFNS protocol, and intraepidermal nerve fibre density of ⩽7.63 fibres/mm on skin sample examination. Such a composite definition has been previously proposed for use in the study of other small fibre neuropathies, but not in HIV-SN [20].

### 2.9 Sample size

At the time of the study there were few data available regarding the diagnostic value for HIV-SN for each of the 2 primary measurements of interest (QST and IENFD) on which to base a sample size calculation. Nevertheless, such a calculation in relation to these measures has been performed in order to guide study conduct (*t* test for power calculation, Sigma v 3.5, Systat Software Inc.).

For QST, the ΔWDT data for HIV positive patients verses healthy controls from Martin et al. [41] were used. This calculation revealed a minimum sample size of 11 was required per group for a power of >0.8 (power 0.828; difference in means 4.3; standard deviation 3.3; α = 0.05).

For IENFD, data for patients with small fibre neuropathies vs healthy controls from Nebuchennykh et al. [46] were used. This calculation revealed a minimum sample size of 16 was required per group for a power of >0.8 (power 0.816; difference in means 4.7; standard deviation 4.5; α = 0.05).

However, because of the uncertain assumptions inherent in such calculations, a minimum group size of 25 was used.

## 3 Results

### 3.1 Participants

Between July 7, 2009, and January 25, 2011, a total of 66 HIV-positive subjects participated in the study. All were ambulatory patients attending the St Stephen’s Centre, Chelsea and Westminster Hospital, London. All 66 participants attended the clinical assessment, but for 2 participants (one from the HIV-SN group and the other from the HIV–No SN group) thermal parameters were missing from the QST data set as a result of an isolated equipment malfunction. Data from these subjects are excluded from the thermal QST data analysis. No participants were excluded by the criterion of pain scored as ⩾4 of 10 on an NRS from a cause other than SN.

IENFD were determined on 57 participants out of the total 66. Six participants did not consent to undergo skin biopsy (4 from the HIV–No SN group and 2 from the HIV-SN group), and 1 participant from the HIV-SN group was receiving anticoagulation therapy (warfarin), and thus skin biopsy was contraindicated. Two samples (1 from each group) were of insufficient quality to allow IENFD determination. Participants with missing IENFD data were not included in the IENFD data analysis.

### 3.2 Demographics

Participant demographic data are presented in Table 1. The HIV–No SN and HIV-SN groups were evenly matched in terms of demographic and related clinical factors. The majority of participants were white (86.4%), male (86.4%), and middle-aged (mean ± SD age, 49.2 ± 8.8 years), broadly reflecting the patient population of the recruitment centre in the current cART era (Dr Marta Boffito and Dr David Asboe, personal communication). Using the triumvirate criteria for HIV-SN diagnosis that we established, the study group was divided into HIV-SN and HIV–No SN groups; 28 participants (42.4%) were thus allocated to the HIV-SN group and 38 (57.6%) to the HIV–No SN group. Twenty-one of the 28 (75.0%) HIV-SN patients reported persistent pain in a distal symmetrical anatomical distribution consistent with a neuropathy. There was no significant difference between the HIV-SN and HIV–No SN groups in terms of sex, ethnicity, height, weight, age, or years since HIV diagnosis. A potential limitation of using the triumvirate is that each of the factors used in the case definition was also examined in the profiling. We examine this further below, and we provide a post hoc analysis of the impact of each factor on participant group allocation.

**Table 1:** Demographics, characteristics, and comorbidities of patients in HIV-SN and HIV–No SN groups.^a^

Characteristic	HIV–No SN (*n* = 38)	HIV-SN (*n* = 28)	*P*
Age, y	47.7 ± 8.9	51.3 ± 8.4	.097
Male	32 (84.2 %)	25 (89.3%)	.553
Height, cm	175.1 ± 8.8	177.1 ± 7.8	.321
Weight, kg	77.1 ± 15.1	80.5 ± 12.2	.334
Years since HIV diagnosis	14.7 ± 7.8	17.8 ± 7.0	.094

*Ethnicity*			
White European, %	33 (86.8%)	24 (85.7%)	.553
African origin, %	4 (10.5%)	3 (10.7%)	.553
Asian, %	1 (2.6%)	0 (0%)	.553
Mixed ethnicity, %	0 (0%)	1 (3.6%)	.553

*Comorbidities*			
Type II diabetes^b^	3 (7.9%)	5 (17.9%)	.220
Hepatitis C infection	9 (23.7%)	6 (21.4%)	.829
Hepatitis B infection	3 (7.9%)	5 (17.7%)	.220
Syphilis	6 (15.8%)	4 (14.3%)	.497
Chemotherapy exposure	1 (2.6%)	3 (10.7%)	.174

HIV-SN, HIV sensory neuropathy; HIV–No SN, no HIV sensory neuropathy.

a Continuous data, if normally distributed, were analysed by Student’s *t* test, and mean ± SD are shown. Categorical data were analysed by χ^2^ test of association; values and percentages are shown.

b No participants with type I diabetes.

There was no difference between the 2 groups for the frequency of comorbidities which are associated with other risk factors for peripheral neuropathy (diabetes mellitus, hepatitis B and C, syphilis, or exposure to chemotherapy agents).

### 3.3 QST healthy control participants

Control data from healthy subjects were collected to meet the DFNS requirements for quality assurance and to provide normal values for suprathreshold heat responses. Thirty-six healthy subjects were recruited with a mean ± SD age of 34.60 ± 9.45 years, 21 (58.3%) of whom were male. These participants underwent the DFNS QST protocol, after which the additional suprathreshold heat testing was conducted on each.

### 3.4 History of ARV therapy

The histories of participants’ ARV therapy are presented in Table 2. Only 2 participants (3.0%) had no previous exposure to ARV drugs; both were in the HIV–No SN group. The majority had previous ARV drug exposure and were all currently receiving cART (*n* = 64, 97.0%). No statistical difference was observed between the 2 groups for either individual agents or for classes of agents in relation to exposure or mean years exposed. There was also no difference between the 2 groups for the total number of drug-years for which a patient received a class of agent or the maximum number of consecutive years of exposure to a given drug class (data not shown).

**Table 2:** Comparison of antiretroviral drug therapy use between HIV–No SN and HIV-SN groups.^a^

Characteristic	HIV–No SN (*n* = 38)	HIV-SN (*n* = 28)	*P*
*ARV*			
ARV therapy	36 (94.74%)	28 (100%)	.218
Years receiving ARV therapy	9.90 ± 6.43	11.77 ± 5.90	.361^b^

*dNRTI*			
Ever received dNRTI	20 (52.63%)	20 (71.43%)	.122
Years receiving dNRTI	12.89 ± 2.32	12.63 ± 3.72	.989^b^

*ddC*			
Ever received ddC	4 (10.53%)	6 (21.43%)	.222
Years receiving ddC	14.90 ± 1.68	16.54 ± 1.50	.142

*d4T*			
Ever received d4T	18 (47.37%)	17 (60.71%)	.283
Years receiving d4T	12.02 ± 1.40	12.31 ± 1.77	.577

*ddI*			
Ever received ddI	12 (31.58%)	14 (50.00%)	.130
Years receiving ddI	11.49 ± 2.84	10.81 ± 3.35	.579

*NRTIs*^c^			
Ever received NRTIs	36 (94.74%)	27 (96.43%)	.744
Years receiving NRTIs	9.15 ± 6.45	11.99 ± 4.99	.053

*NNRTIs*			
Ever received NNRTIs	28 (73.68%)	25 (92.59%)	.115
Years receiving NNRTIs	8.17 ± 6.53	10.53 ± 6.53	.192

*PI*			
Ever received PI	21 (55.26%)	21 (75.00%)	.099
Years receiving PI	21.56 ± 14.02	28.55 ± 21.10	.687^b^

*IDV*			
Ever received IVD	5 (13.16%)	9 (32.14%)	.062
Years receiving IDV	13.28 ± 0.68	12.64 ± 1.15	.280

HIV-SN, HIV sensory neuropathy; HIV–No SN, no HIV sensory neuropathy; ARV, antiretroviral therapy; dNRTI, deoxy nucleoside reverse transcriptase inhibitors; ddC, zalcitabine; d4T, stavudine; ddI, didanosine; NNRTI, nonnucleoside reverse transcriptase inhibitors; PI, protease inhibitor; IVD, indinavir.

a Continuous data, if normally distributed, were analysed by Student’s *t* test, and mean ± SD are shown. Continuous data not normally distributed were analysed by Mann-Whitney rank sum test. Categorical data were analysed by χ^2^ test of association; values and percentages are shown.

b Not normally distributed.

c All NRTIs, including dNRTIs.

There was no statistical difference between the 2 groups for exposure to dNRTI agents; 52% (HIV–No SN) and 71% (HIV-SN) of participants had been exposed at some point to neurotoxic dNRTIs (*P* = .122), and the mean exposure was about 12 years. This was independent of time since HIV diagnosis. The groups were also matched for exposure to the potentially neurotoxic protease inhibitor indinavir [12], [22], [49], [62], with 13% (HIV–No SN) and 32% (HIV-SN) of subjects having been exposed to the drug for a mean of about 13 years.

### 3.5 Quantitative sensory testing

**Fig. 1:**
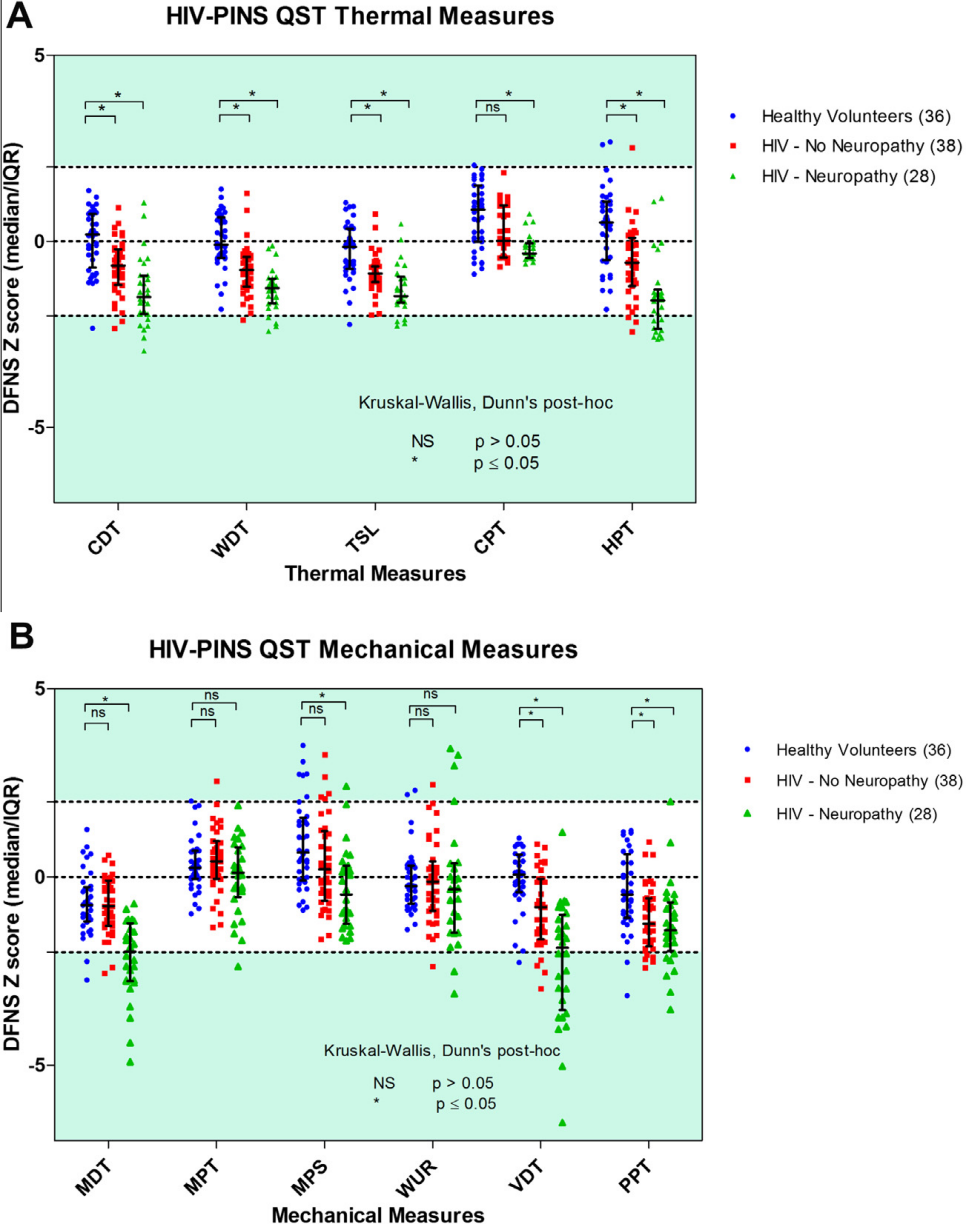
Dot plot of z-score QST parameters in the healthy control, HIV–No SN and HIV-SN groups for Mechanical QST parameters (A) and Thermal QST parameters (B). ^∗^Kruskal-Wallis, Dunn’s post hoc 1-way analysis of variance: ^∗^*P* < .05; NS, *P* > .05. QST, quantitative sensory testing; HIV-SN, HIV sensory neuropathy; HIV–No SN, no HIV sensory neuropathy; NS, not significant.

**Fig. 2:**
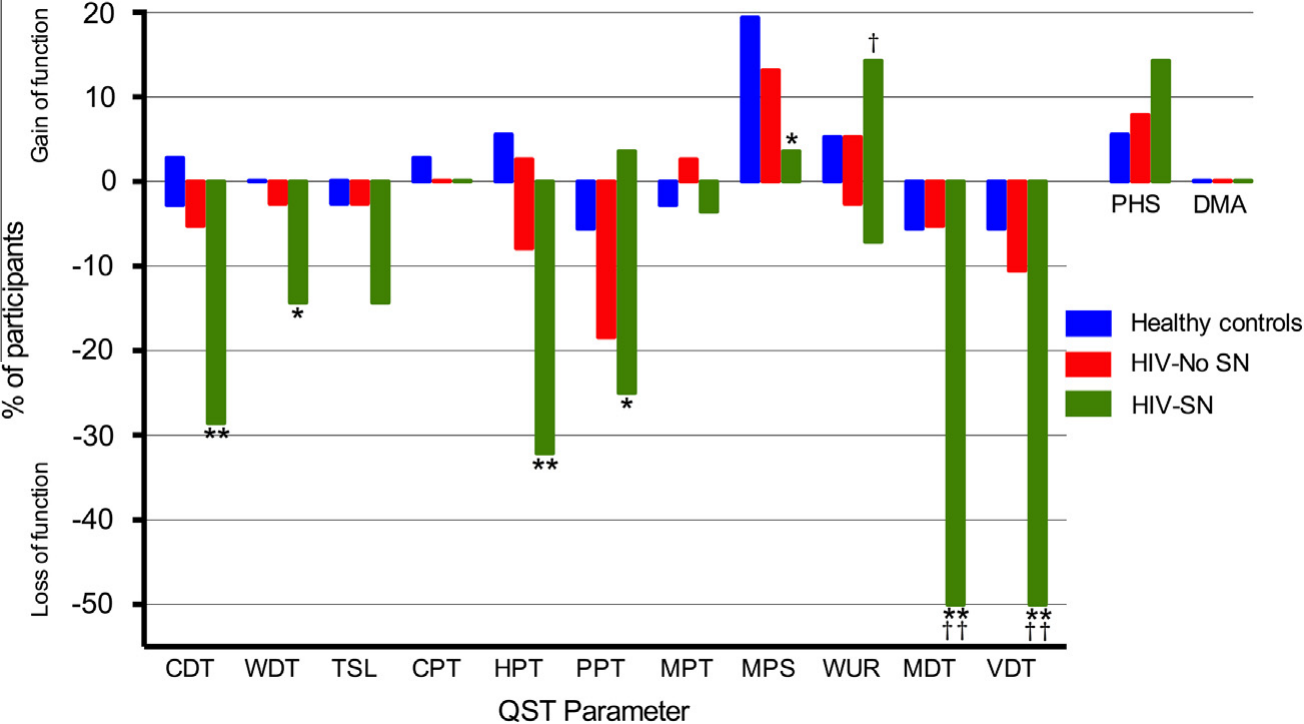
Loss and gain of sensory function. Comparison of participants in the HIV-SN group, HIV–No SN group, and healthy controls who have QST values outside the 95% confidence interval of the DFNS reference database. The *y-*axis shows the percentage of patients in each group (HIV-SN *n* = 38, HIV–No SN *n* = 28, healthy controls *n* = 36), with ‘gain’ of sensory function plotted upwards and ‘loss’ of sensory function plotted downwards. HIV-SN, HIV sensory neuropathy; HIV–No SN, no HIV sensory neuropathy; QST, quantitative sensory testing; HIV–No SN, no HIV sensory neuropathy. Chi squared test of association: ^∗^*P* < 0.05, ^∗∗^*P* < 0.01 comparison to Healthy controls; ^†^*P* < 0.05, ^††^*P* < 0.01 comparison to HIV-No SN.

*Distribution of mean QST z-scores across groups.* The data for thermal QST parameters are presented in Fig. 1 and Supplementary Document 4. The mean z-scores for all thermal parameters fall within the DFNS normative range, although data from individual patients are seen outside the normative range, especially for CDT, WDT, and HPT in the HIV-SN group (Fig. 1). However, between-group comparisons (Fig. 1) reveal significant differences, in the loss-of-function direction, between the locally recruited healthy control groups and the HIV–No SN and HIV-SN groups (*P* < .05, Kruskal-Wallis, Dunn’s post hoc test). This general shift across all thermal parameters towards hypoesthesia was largest in the HIV-SN group. Mean z-score values, when compared to the DFNS normative data set, reveal a loss of function for MDT and VDT in the HIV-SN group (Supplementary Document 4). No such effect was observed for the other mechanical sensory parameters. This loss of sensation was also reflected when data for these parameters were compared with those obtained from locally recruited healthy controls (Fig. 2; *P* < .05, Kruskal-Wallis, Dunn post hoc test). Supplementary Document 4 shows that mean healthy control QST participant data closely match that predicted by DFNS control values for 12 of the 13 DFNS QST parameters. This is indicated by the z-transformed QST mean values in the healthy control group all being close to 0, with SDs of near to 1 or less. MPS values in healthy control subjects showed a slight shift in mean values compared to DFNS data, with 5 (13.9%) participants showing abnormal values compared to DFNS healthy control data.

*Frequency of individual QST measure abnormalities: loss and gain of sensory function.* The frequency of abnormal QST values in each group are shown in Fig. 2 and Supplementary Document 4. The majority of abnormal parameters were loss of sensory function for any of the QST parameters, with 34.2% and 85.7% of participants in the HIV–No SN and HIV-SN groups, respectively, displaying evidence of at least 1 abnormal value. There was no difference between the groups in the number of participants in whom gain-of-function phenomena were observed: 26.3% in HIV–No SN, 28.6% in HIV-SN, and 30.6% of healthy controls. The most frequent sensory abnormalities seen in the HIV-SN group were loss of MDT and VDT, both of which were independently abnormal in 50.0%, with 32.14% demonstrating the loss of both in the HIV-SN group. In contrast, in the healthy control group and the HIV–No SN group, sensory loss for MDT was found in only 5.6% and 5.3%, and for VDT in 5.6% and 10.5%, respectively.

Within thermal sensory parameters, the HIV-SN participants showed a loss of CDT function (28.6%) most frequently (compared to 5.3% in the HIV–No SN group), followed by WDT and TSL (14.3% and 2.6% in both HIV groups). Loss of HPT was the most frequent abnormal thermal nociceptive parameter in the HIV-SN group (32.1%) and in the HIV–No SN group (7.9%). No participant in either HIV group displayed abnormalities of CPT. The presence of PHS was considered a loss of thermal discrimination and therefore a loss of sensory function [57]. PHS phenomena were observed in 7.9% and 14.3% of the HIV–No SN and HIV-SN groups, respectively.

The presence of gain-of-sensory function was rare across all groups but was seen most frequently in the mechanical WUR parameter, with 14.3% of HIV-SN patients demonstrating it compared to only 5.3% and 5.6% of the healthy control and HIV–No SN groups (*P* < .05, χ^2^ test of association against HIV–No SN group). Examination of the dot plots in Fig. 1 reveals a potential subset of 4 HIV-SN participants who are characterised by high (gain of function) z-scores for WUR (*P* < .05, χ^2^ test of association compared to HIV–No SN group). No participants in any of the groups were found to have DMA.

*Patterns of loss and gain of sensory function.* HIV-SN-associated abnormalities in QST parameters were dominated by loss of sensory function effects (Table 3). Indeed, the majority of HIV-SN participants (85.7%) had at least 1 abnormality on QST testing and had, at a minimum, loss of 1 sensory parameter. Different combinations of sensory loss and gain did occur across the HIV-SN group; however, the majority involved just sensory loss (L1 + L2 + L3 = 78.6%). The most frequent combination was the loss of at least 1 thermal and 1 mechanical QST parameter in 42.9% of participants (L3G0). HIV–No SN participants demonstrated loss or gain of thermal modalities in isolation (L1 or G1) (Table 3). The second most frequent combination was loss of just mechanical sensory modalities (L2G0, 21.4%).

**Table 3:** Frequency of different patterns of sensory loss and gain in HIV-SN group (*n* = 28).

Characteristic	Gain 0	Gain 1	Gain 2	Gain 3	Gain, all
Loss 0	4 (14.3%)	0	0	0	4 (14.3%)
Loss 1	0	0	0	0	0
Loss 2	6 (21.4%)	0	4 (14.3%)	0	10 (35.7%)
Loss 3	12 (42.9%)	0	2 (7.1%)	0	14 (50%)
Loss, all	22 (78.6%)	0	6 (21.4%)	0	28 (100%)

Loss 0, no loss of detection; Loss 1, loss of only thermal loss; Loss 2, loss of only mechanical; Loss 3, loss of both thermal and mechanical; Gain 0, no gain of detection; Gain 1, gain of only thermal; Gain 2, gain of only mechanical; Gain 3, gain of thermal and mechanical.

**Fig. 3:**
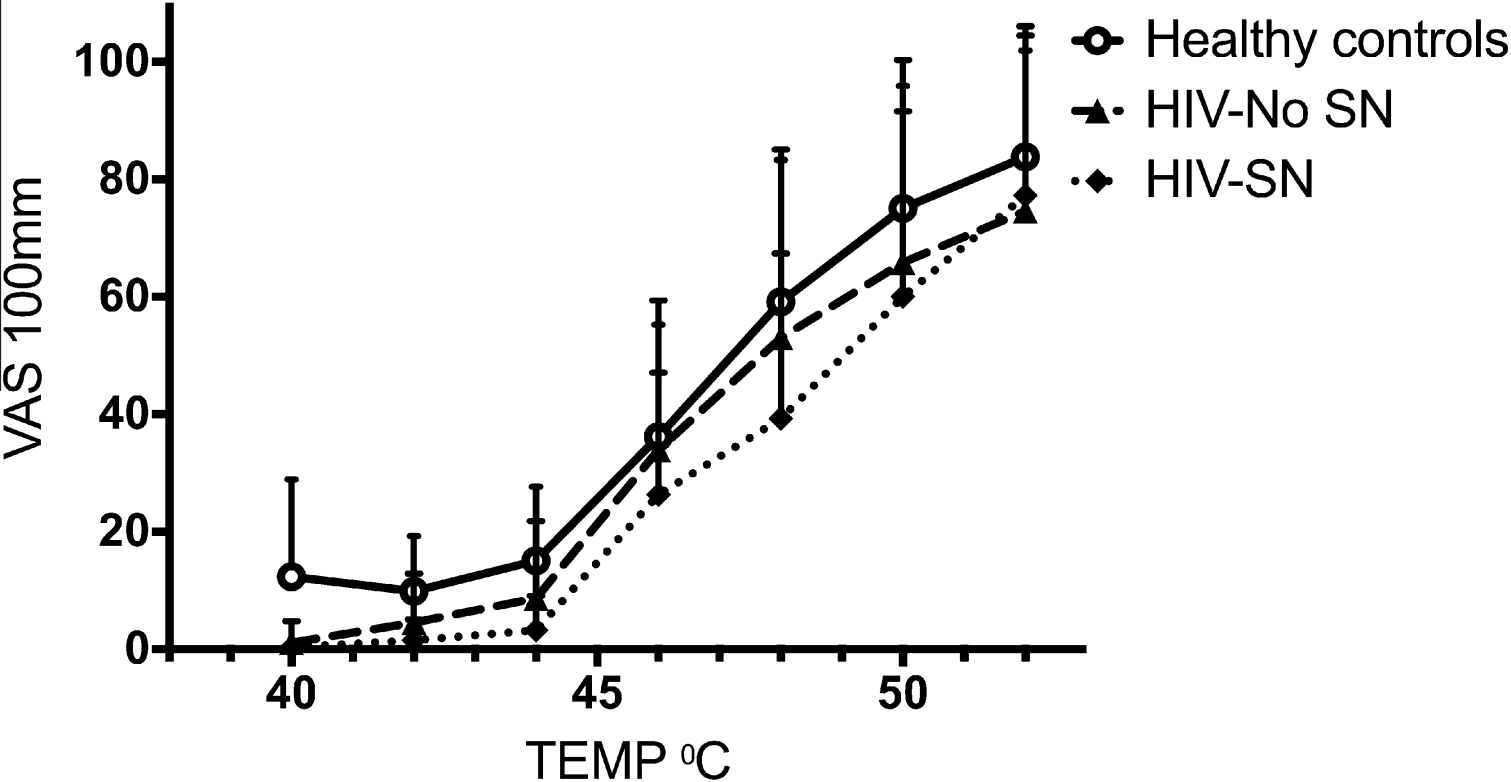
Pain intensity response curves for suprathreshold heat stimuli in healthy control subjects (*n* = 36), HIV–No SN group (*n* = 38), and HIV-SN (*n* = 28) group. Data are presented as mean (SD). No statistically significant difference was present between groups. VAS, visual analogue scale; HIV-SN, HIV sensory neuropathy; HIV–No SN, no HIV sensory neuropathy.

*Suprathreshold heat testing.*Fig. 3 displays the cumulative mean pain intensity responses to suprathreshold heat stimuli in the range between 48°C and 52°C for the 3 groups: healthy controls, HIV–No SN group, and HIV-SN group. Healthy control patients had an earlier take-off point in pain visual analogue scale (VAS) responses—that is, this occurred at lower temperatures. However, at higher suprathreshold temperatures, there was little difference in the VAS responses elicited from all the 3 groups tested. No statistically significant difference was seen between the 3 groups or in the painful HIV-SN subgroup of HIV-SN (data not shown) for pain response at any suprathreshold temperature.

### 3.6 Intraepidermal fibre densities

**Fig. 4:**
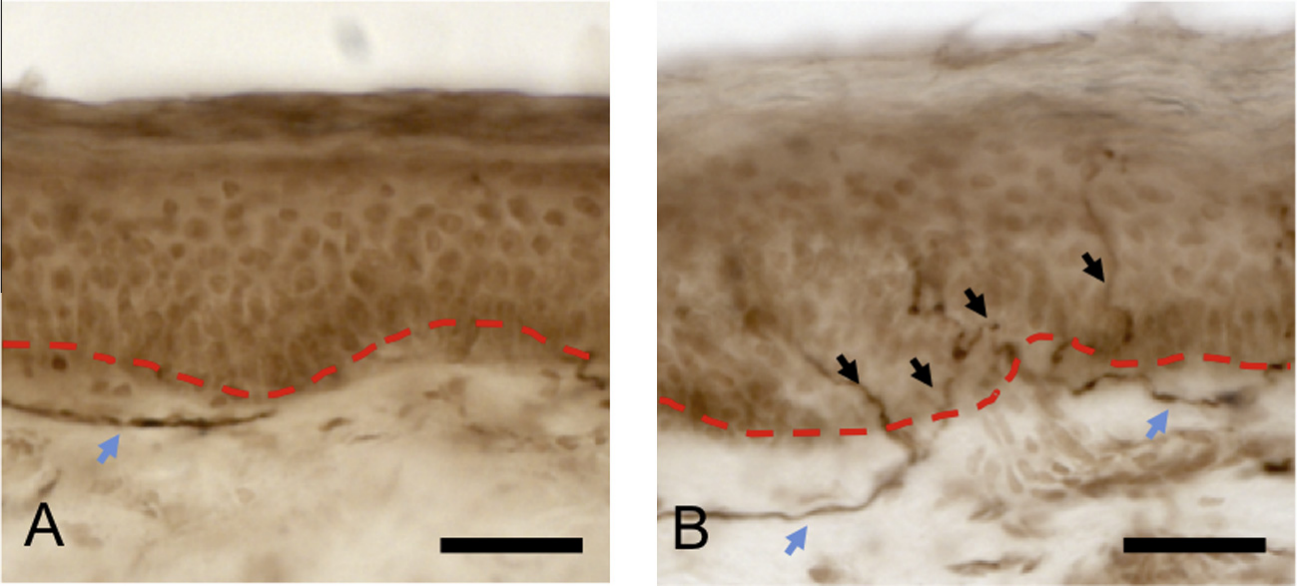
Two representative skin biopsy samples of HIV-infected participants. (A) Participant from HIV-SN peripheral neuropathy group demonstrating complete absence of small unmyelinated sensory nerve fibres reaching epidermis. Subepidermal dermal plexus fibres are present (blue arrow) as identified by pan neuronal marker PGP 9.5. (B) Participant from HIV–No SN group with normal counts of small unmyelinated nerve fibres (black arrows) reaching epidermis beyond dermal epidermal junction (red dotted line), and positive dermal plexus staining (blue arrows). Original magnification, 40×. Scale bar = 50 μm. HIV-SN, HIV sensory neuropathy; HIV–No SN, no HIV sensory neuropathy.

A total of 57 skin punch biopsy samples were available for IENFD measurement from the 66 participants. Fig. 4 shows representative images of 2 participants, one with reduced IENFD counts (Fig. 4A) and the other with normal fibre counts (Fig. 4B).

The associated intraobserver correlation coefficients for 20 randomly selected samples for quality assurance exercise was 0.88 (JDR) and 0.89 (MB), both of which are high. A high interobserver Pearson’s product-moment correlation coefficient of 0.93 was determined between the 2 microscopists (JDR and MB) for all (*n* = 55) study samples.

The median values of IENFD for HIV–No SN group was 9.2 fibres/mm (range 1.7–14.4 fibres/mm) compared to 6.3 fibres/mm (range 0.7–12.4 fibres/mm) for the HIV-SN group (*P* < .001). In accordance with previously published literature, an IENFD value of <7.63 fibres/mm was taken as being abnormal [35]; this was seen in 8 (21.1%) of the HIV–No SN group and 17 (60.7%) in the HIV-SN group (*P* < .05), with an associated sensitivity and specificity of detecting HIV-SN in isolation of 61% and 79%. This result might be expected, given the case definition used.

Supplementary Document 5 provides Pearson’s correlation coefficients for individual QST parameters and IENFD. No correlation between the measured QST parameters and IENFD was observed.

As would be expected, IENFD was inversely correlated to the severity of the peripheral neuropathy as measured by the TCSS instrument (*r* = −0.343, 95% confidence interval −0.56 to −0.88, *P* < .01).

### 3.7 Pain and patient-reported symptoms

Results of the structured patient symptom and medical history interview, and the 7-day pain diary are presented in Table 4.

**Table 4:** Comparison of participant reported symptoms between HIV-SN and HIV–No SN groups.^a^

Reported symptom	HIV–No SN (*n* = 38)	HIV-SN (*n* = 28)	*P*
Any pain in hands and/or feet	11 (28.95%)	21 (75.00%)	<.001
If experiencing pain: 7-d pain diary, NRS (0–10)	2.81 ± 2.34	5.65 ± 1.76	<.001
Pain onset, y after HIV diagnosis	12.29 ± 5.94	9.50 ± 7.59	.358
Pain duration, y	9.50 ± 7.59	8.25 ± 7.23	.811
‘Pins and needles’ in feet and/or hands	19 (50.00%)	17 (60.71%)	.388
‘Numbness’ in feet and/or hands	14 (36.84%)	21 (75.00%)	<.02
Perceived ‘weakness’ in upper or lower limbs	10 (26.32%)	10 (35.71%)	.412
Postural hypotension	9 (24.68%)	14 (50.00%)	<.02
Urinary dysfunction	6 (15.79%)	12 (42.86%)	<.02
Erectile dysfunction	12 (31.58%)	14 (50.00%)	.152
Nocturnal diarrhoea	10 (26.32%)	8 (28.57%)	.839

HIV-SN, HIV sensory neuropathy; HIV–No SN, no HIV sensory neuropathy; NRS, numerical rating scale.

a Continuous data, if normally distributed, were analysed by Student’s *t* test, and mean ± SD are shown. Categorical data were analysed by χ^2^ test of association; values and percentages are shown.

More participants reported pain in their hands and/or feet in the HIV-SN group compared to HIV–No SN group (75.00% vs 28.95% respectively, *P* < .001). The mean ± SD 7-day pain intensity score for patients experiencing pain was also greater (*P* < .001) in the HIV-SN group (5.65 ± 1.76) compared to the HIV–No SN group (2.8 ± 2.34). The mean number of years from HIV diagnosis and the mean duration of pain were similar in the 2 groups. Although there was no difference between groups in the reporting of ‘pins and needles’, the reporting of ‘numbness’ in feet and/or hands was more frequent in the HIV-SN group (75.0%) compared to the HIV–No SN group (36.8%, *P* < .05).

### 3.8 NPSI

**Fig. 5:**
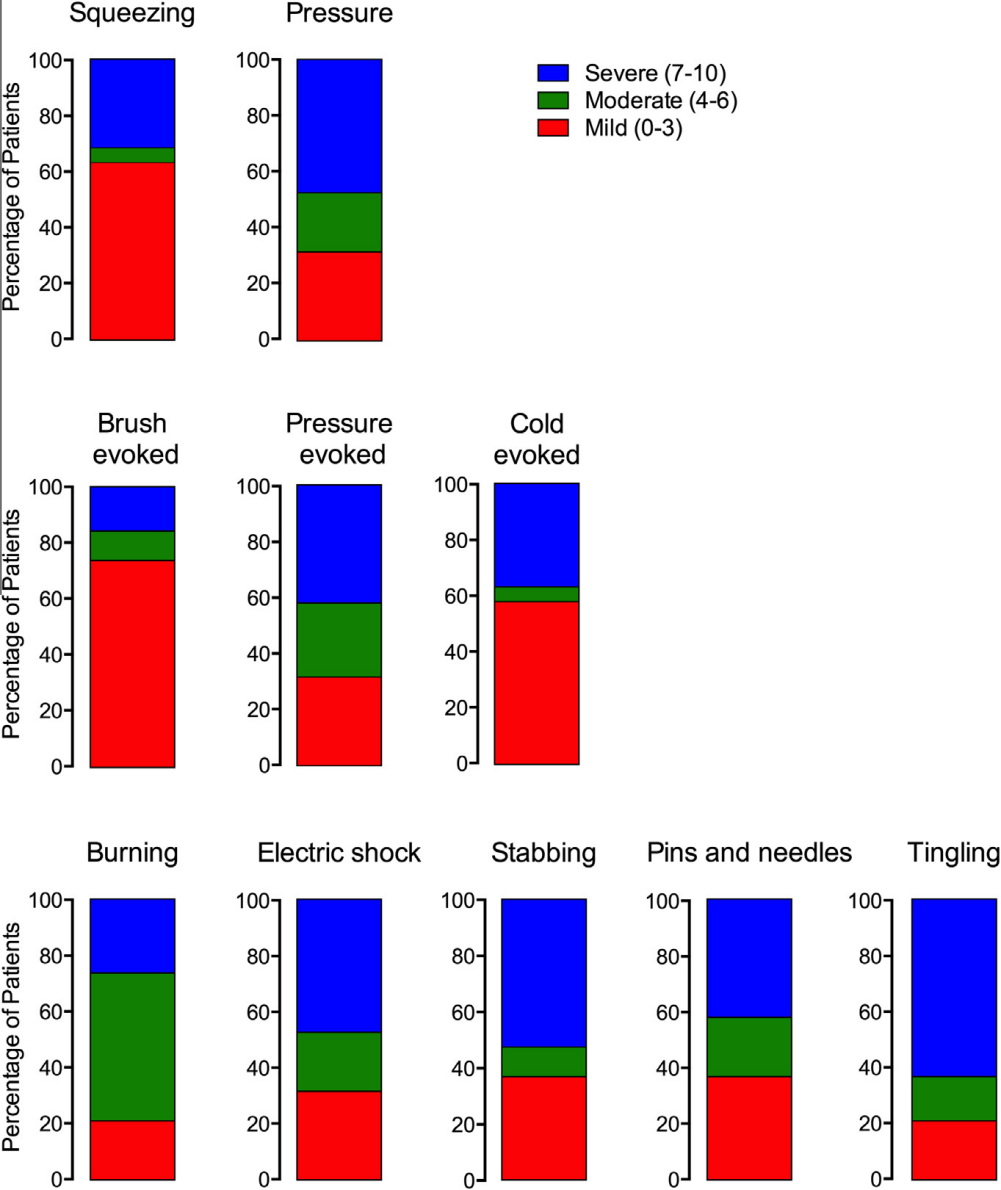
Distribution of NPSI descriptors in the painful HIV-SN population divided by categories of severity. NPSI, Neuropathic Pain Symptom Inventory; HIV-SN, HIV sensory neuropathy.

Individual participant responses to the 10 NPSI items were divided into mild, moderate, and severe and are shown in Fig. 5 for participants experiencing painful HIV-SN. Mean values are shown in Supplementary Document 6.

Of the 7 nonevoked pain items, more than 50% of participants reported moderate to severe symptoms for all of these, except ‘squeezing pain’ (36%). ‘Burning’ was the most frequently reported as being moderate or severe; however, ‘tingling’ was the most frequently reported as being severe (63%).

Of the 3 evoked pain items, ‘pressure evoked’ was most often reported as being moderate or severe (68%), followed by ‘cold evoked’ (42%) and ‘brush evoked’ (26%).

No correlations were seen between the descriptive NPSI scores and QST values.

### 3.9 Autonomic function

More participants with HIV-SN compared to those without HIV-SN, reported experiencing symptoms of postural hypotension (50.0% vs 24.6% respectively, *P* < .01); however, participants’ reported symptoms of orthostatic hypotension correlated to measured orthostatic hypotension in only 33.3% of HIV–No SN and 7.1% of HIV-SN participants. No difference was seen in measured orthostatic hypotension between the 2 groups (14.8% vs 18.4% for HIV-SN and HIV–No SN groups, respectively; *P* = .702).

Patients reported symptoms of urinary dysfunction more frequently in the HIV-SN group (42.9%) compared to the HIV–No SN group (15.8%, *P* < .05). The frequency of participants reporting erectile dysfunction and nocturnal diarrhoea were similar for both groups (Table 4).

### 3.10 Psychological problems, sleep disturbance, and health-related quality of life

Results from the psychological and insomnia instrument battery are presented in Table 5. Of the 66 participants, 57 (86.3%) returned completed questionnaire booklets to the test centre for analysis. The return of questionnaires and completion of individual scores within each group are shown in Supplementary Document 7. We had planned a comparison of HIV-SN without pain and HIV-SN with pain to the participants without neuropathy (HIV–No SN). However only a small number of participants with HIV-SN with no pain (7 individuals) were found, making a comparison unlikely to be meaningful. These individuals were not included (as part of the HIV-SN group) in the analysis of psychological instruments, insomnia, or health-related quality of life, as their presence could potentially confound the influence of pain in the HIV-SN group when comparing it to the HIV–No SN group.

**Table 5:** Summary of sleep and psychological instruments results comparing HIV–No SN and painful HIV-SN.

Instrument	HIV–No SN (*n* = 38)	HIV-SN with pain (*n* = 21)	*P*
ISI			
Mean ISI total score (/28)	10.16 ± 7.86	14.05 ± 8.86	.112
Participants with clinical insomnia (ISI ⩾15)	7 (22.6%)	13 (68.4%)	<.001
Participants with severe insomnia (ISI ⩾22)	3 (9.7%)	7 (22.6%)	<.001

DAPOS			
DAPOS depression (/25)	8.38 ± 4.10	11.21 ± 4.22	<.05
DAPOS anxiety (/15)	5.45 ± 2.89	7.47 ± 2.97	<.05

PASS-20			
PASS cognitive (/25)	8.45 ± 5.28	14.95 ± 5.69	<.001
PASS escape-avoidance (/25)	9.07 ± 7.15	11.84 ± 6.38	.177
PASS fear (/25)	6.97 ± 7.11	11.79 ± 6.75	<.05
PASS physiological anxiety (/25)	4.83 ± 6.45	9.21 ± 7.54	<.05
PASS total (/100)	35.84 ± 29.84	47.79 ± 21.94	.136

PCS			
PCS rumination (/16) 5.68 ± 4.85	8.84 ± 5.11	0.330	
PCS magnification (/12)	3.97 ± 3.66	5.63 ± 3.39	.115
PCS helplessness (/24)	6.03 ± 5.52	11.16 ± 6.131	<.02
PCS total (/52)	14.13 ± 11.81	23.74 ± 12.64	<.02

BPI			
BPI interference (/70)	15.20 ± 16.22	46.11 ± 13.69	<.001

SF-36			
Physical Functioning (PF)	74.03 ± 24.65	34.47 ± 21.74	<.001
Role Physical (RP)	50.78 ± 43.76	11.84 ± 28.10	<.001
Bodily Pain (BP)	69.43 ± 24.47	29.42 ± 18.06	<.001
General Health (GH)	43.78 ± 26.96	26.58 ± 19.88	<.05
Vitality (VT)	49.53 ± 24.08	25.79 ± 24.45	<.001
Social Functioning (SF)	67.98 ± 27.31	32.24 ± 24.41	<.001
Role Emotional (RE)	54.17 ± 46.18	15.79 ± 32.14	<.05
Mental Health (MH)	63.25 ± 21.25	47.79 ± 17.05	<.05

HIV-SN, HIV sensory neuropathy; HIV–No SN, no HIV sensory neuropathy; ISI, Insomnia Severity Index; DAPOS, Depression Anxiety Positive Outlook Scale; PASS-20, Pain Anxiety Symptom Scale Short Form; PCS, Pain Catastrophizing Scale; SF-36, Short Form (36) Health Survey.

### 3.11 Pain interference and health-related quality of life

As expected, the BPI interference subscale mean total score was significantly higher in the painful HIV-SN group compared to the HIV–No SN group. The high scores present in the painful HIV-SN group indicate that participants’ pain is associated with a detrimental impact on their day-to-day living and their quality of life.

The SF-36 data further establish that participants with painful HIV-SN experienced significantly more difficulties compared to the HIV–No SN group across all of the domains of the SF-36 (Table 5). In particular, HIV-SN patients’ physical functioning, perceptions of their physical selves, general vitality, and social functioning are poorer in the painful HIV-SN group compared to patients in the HIV–No SN group.

### 3.12 Sleep dysfunction

Analysis of data from the self-report ISI instrument indicated that both patient groups on average experienced mild subclinical insomnia; at the group level, there was no independent effect of HIV-SN. At the individual level, more participants in the painful HIV-SN group reported sleep disturbance equating to clinical insomnia (ISI ⩾ 15); almost twice as many participants in the HIV-SN group experienced severe insomnia (ISI ⩾ 22).

### 3.13 Depression, anxiety, and catastrophizing

Participants with painful HIV-SN were more depressed and anxious as measured by the DAPOS than those in the HIV–No SN group (Table 5).

Overall, there was no difference between the groups with regard to the total PASS-20 scores (Table 5). However, differences were evident in the specific domains of the PASS-20. The HIV-SN group reported more features of cognitive impairment in response to pain compared to the HIV–No SN group. There was more physiological anxiety and fear as measured with the PASS-20 subscales in the painful HIV-SN group compared to the HIV–No SN group.

There was a difference between the groups for catastrophizing (Table 5): mean ± SD 14.3 ± 11.81 in the HIV–No SN group compared to 23.74 ± 12.64 in the painful HIV-SN group (*P* = .009). This difference was largely attributable to a difference in the PCS helplessness subscale.

### 3.14 Plasma lipid profiles, random glucose, body mass index, and waist–hip circumference ratios

A higher mean triglyceride plasma (TRG) concentration was measured in the HIV-SN group 2.18 ± 1.09 mmol/L compared to that for the HIV–No SN group, at 1.61 ± 0.77 mmol/L (*P* < .05; Table 6). There was no significant difference between the 2 groups in statin drug use. Despite this difference in TRG concentrations, we did not find a correlation between the severity of neuropathy (using the TCSS instrument) and plasma TRG concentrations (*r_s_* = −0.155, 95% CI [−0.514 to 0.250], *P* = .439). No significant differences were found between the 2 groups for other plasma lipids, body mass index, or waist–hip circumference ratios, although all were on the upper end of normal (Table 6).

**Table 6:** Comparison of various metabolic factors in HIV–No SN and HIV-SN groups.^a^

Metabolic factor	HIV–No SN (*n* = 38)	HIV-SN (*n* = 28)	*P*
Total cholesterol, mmol/L	4.56 ± 1.08	5.01 ± 1.07	.100
Triglycerides, mmol/L	1.61 ± 0.77	2.18 ± 1.09	<.020^b^
HDL, mmol/L	1.06 ± 0.36	1.24 ± 0.44	.069
LDL, mmol/L	2.75 ± 0.93	2.71 ± 0.95	.860
Cholesterol:HDL ratio	4.53 ± 1.57	4.42 ± 1.27	.769
Random glucose, mmol/L	5.31 ± 1.20	5.31 ± 1.07	.863^b^
BMI, kg/m^2^	25.28 ± 5.34	25.68 ± 3.69	.223^b^
Waist–hip circumference ratio	0.98 ± 0.09	0.96 ± 0.12	.739^b^
Current statin use	9 (23.70%)	9 (32.14%)	.446

HIV-SN, HIV sensory neuropathy; HIV–No SN, no HIV sensory neuropathy; HDL, high-density lipoprotein; LDL, low-density lipoprotein; BMI, body mass index.

a Continuous data, if normally distributed, were analysed by Student’s *t* test, and mean ± SD are shown. Continuous data not normally distributed were analysed by Mann-Whitney rank sum test. Categorical data were analysed by χ^2^ test of association; values and percentages are shown.

b Not normally distributed.

### 3.15 Peripheral neuropathy screening and severity instruments: BPNS, UENS, and TCSS

Mean scores were determined and receiver operating characteristic (ROC) plots generated for each of the 3 neuropathy screening instruments examined in the study. Optimal cutoff values for the diagnosis of HIV-SN in the study population were determined from these plots, giving equal weight for sensitivity and specificity.

Using the BPNS scoring method described by Cherry et al. [14] (which weights loss of deep tendon reflexes and vibration perception measures), the BPNS instrument has a specificity and sensitivity in the detection of HIV-SN of 75% and 79% (*P* < .001). When the numerical component of the BPNS is used in its raw form, weighting sensitivity and specificity equally, a cutoff value of 19 is associated with sensitivity and specificity of 76%.

The mean ± SD TCSS values for HIV–No SN compared to HIV-SN were 3.36 ± 3.65 and 9.26 ± 3.28, respectively (*P* < .001). ROC analysis found a value of 8 provided the best accuracy in HIV-SN detection; sensitivity and specificity were 79%.

The UENS median ± SD values for HIV–No SN compared to HIV-SN group were 3.64 ± 2.98 and 11.26 ± 5.66, respectively (*P* < .001). The original validation study for the UENS did not suggest a cutoff score for the diagnosis of a peripheral neuropathy; giving equal weight to sensitivity and specificity, a value of 7 was selected in this current study. This provides a sensitivity and specificity of 84% in detection of HIV-SN.

Using the ROC plots for each instrument, area under the curve (AUC) was used as a measure of accuracy, with a larger AUC equating to greater accuracy. The instrument found to have the greatest accuracy was the UENS (AUC 0.91), followed by TCSS (AUC 0.86) and then the BPNS (AUC 0.69).

### 3.16 Testing the HIV-SN triumvirate definition

As this study’s case definition of HIV-SN used a triumvirate utilising QST, IENFD and a SNE, we tested the consequences of using different combinations of the triumvirate on the HIV-SN designation of participants in the study (Table 7). Removal of QST and IENFD individually from the triumvirate resulted in sensitivity and specificity of 71%, 100% and 79%, and 100%, respectively, when compared to using the full triumvirate diagnosis. Not using QST in the diagnosis resulted in 8 (12.1%) participants having a different diagnosis; not using IENFD results in a change in 6 (9.1%); and removal of SNE resulted in the largest number of diagnosis changes, at 17 (25.8)%.

**Table 7:** Comparison of the use of different combinations of the triumvirate criteria for diagnosis of HIV-SN.^a^

Changed diagnosis	QST + CNE + IENFD	QST + CNE	QST + IENFD	CNE + IENFD	QST	CNE	IENFD
‘HIV–No SN’ changed to ‘HIV-SN’, *n* (%)	0 (0%)	0 (0%)	0 (0%)	0 (0%)	7 (18.42%)	10 (26.32%)	8 (21.05%)
‘HIV-SN’ changed to ‘HIV–No SN’, *n* (%)	0 (0%)	6 (21.43%)	17 (60.07%)	8 (28.57%)	6 (21.43%)	0 (0%)	11 (39.29%)
No. changed (% of total participants)	0 (0%)	6 (9.09%)	17 (25.76%)	8 (12.12%)	13 (19.70%)	10 (15.15%)	19 (28.79%)
Sensitivity^b^	1.000	0.786	0.393	0.714	0.786	1.000	0.607
Specificity^b^	1.000	1.000	1.000	1.000	0.816	0.740	0.790

HIV-SN, HIV sensory neuropathy; HIV–No SN, no HIV sensory neuropathy; QST, quantitative sensory testing criteria; CNE, clinical neurological examination; IENFD, intraepidermal nerve fibre density criteria.

a The case definition used in the study required the presence of 2 or more out of the following triumvirate: clinical signs of distal sensory neuropathy, 2 or more abnormal QST parameters, or IENFD of ⩽7.63 fibres/mm (QST + CNE + IENFD). Each column shows the consequences of changing the case definition.

b Sensitivity and specificity compared to use of triumvirate of QST + CNE + IENFD criteria for diagnosis of HIV-SN.

The criteria most effective in isolation is SNE, with a sensitivity and specificity of 1.00 and 0.740, followed by QST (sensitivity 0.786, specificity 0.816) and IENFD (sensitivity 0.607, specificity 0.790).

## 4 Discussion

In this first report of the detailed HIV-SN phenotype in the cART era, the predominant sensory feature was loss of function; 86% of HIV-SN participants had loss of function in at least 1 sensory modality. However, the degree to which individual modalities were affected across the HIV-SN group did not correlate with either symptoms (including pain) or neuropathy severity. No single sensory parameter alone has diagnostic utility for HIV-SN.

The main sensory finding was loss of function of the Aβ fibre-mediated sensory modalities of mechanical and vibration detection thresholds. HIV-SN is usually described as a SFN; however, assessment of small fibre function using thermal detection thresholds did not detect abnormalities to a sufficient extent to yield diagnostically useful information, in contrast to the IENFD measures. Similarly, no diagnostic utility was seen in heat suprathreshold stimulus response testing. This is a similar finding to that seen in a pre-cART-era study [8].

Although this was a small sample, 4 HIV-SN patients with pain (14%) had increased WURs associated with raised catastrophizing scores, of 26.0 (interquartile range 22.5–31.5) vs 12.0 (interquartile range 0–22.0) (*P* < .05, Mann-Whitney rank sum test). Although this sample is too small to draw conclusions, high WUR and raised PCS have been related in phantom limb pain [65]. This merits further study in a larger sample.

### 4.1 Intraepidermal nerve fibre density

IENFD counts were lower in the HIV-SN group, and the severity of neuropathy, using the TCSS, was inversely correlated to IENFD. However, the diagnostic utility of counts <7.63 fibres/mm can only be considered moderate in isolation, with a sensitivity and specificity of 61% and 79%, respectively, of detecting HIV-SN compared to the study’s diagnostic triumvirate. Consistent with other reports, no correlation was found between IENFD and sensory nerve fibre dysfunction for individual QST parameters [17], although contrasting findings have been reported [59].

After recruitment had started, the guidelines for skin biopsy-based diagnosis of small fibre neuropathies were updated [36] to recommend the use of sex/age-matched control data [3]. However, the diagnostic utility of age/sex-matched normal values have been compared [46]. Age/sex-adjusted cutoff values produced the best specificity (98%) but had low sensitivity (31%); a cutoff value of 10.3 fibres/mm produced poorer specificity (64%) but improved sensitivity (78%). The lower cutoff value of 7.63 fibres/mm [20] used in this study is associated with a specificity of 90% and sensitivity of 80% and represents an appropriate compromise between the age/sex-matched and ROC methods*.*

We used the per protocol criterion of 7.63 fibres/mm as the cutoff value in the triumvirate HIV-SN definition. Nevertheless, a post hoc analysis using the 2010 guidelines was performed using the recommended normative values. This analysis demonstrated no change in HIV–No SN group allocation; however, 5 participants (8%) originally allocated to HIV-SN were now allocated to HIV–No SN. This was thought to be acceptable because the difference is proportional to what would be expected using this more conservative definition of HIV-SN. Additionally, 3 of the 5 participants whose diagnoses were altered to HIV–No SN reported painful peripheral neuropathy symptoms, suggesting that a diagnosis of HIV-SN was likely to be correct for these participants.

### 4.2 Metabolic factors

Higher plasma TRG concentrations were measured in the HIV-SN group compared to the HIV–No SN group. This finding underlines the emerging understanding that dyslipidaemia is important in the development of HIV-SN [2], [4] and other peripheral neuropathies [66], [71]. There was no difference in statin use between the 2 groups. The role of other metabolic factors in this study was less clear.

### 4.3 Psychology, sleep dysfunction, and pain symptomatology

Participants with painful HIV-SN show interference from pain on the BPI and reduced quality of life compared to participants without neuropathy across most domains of the SF-36. Findings were comparable to changes in quality of life in other painful peripheral neuropathies, but HIV-SN appears to be associated with greater disability [53] and poorer overall perception of general health. Pain-related anxiety symptoms were similar to other pain conditions across most domains [18] but with less report in HIV-SN patients of escape/avoidance behavior.

Sleep laboratory and self-report insomnia data have been reported in HIV infection [56]. However, few studies have examined the role of pain or neuropathy in HIV-associated insomnia. We have shown a higher incidence of insomnia in the painful HIV-SN group compared to the HIV–No SN group. Other forms of neuropathic pain are also associated with coincident insomnia [34].

The NPSI participants with painful HIV-SN reported experiencing multiple neuropathic pain symptoms; however, burning, tingling, pressure pain, and pressure-evoked pain were the most frequently items to be reported as ‘moderate’ or ‘severe’. There was no correlation between NPSI and QST parameters, which is similar in other painful sensory neuropathies [24].

### 4.4 Diagnostic tools

The use of a robust composite HIV-SN definition as used herein would prove time-consuming and expensive for use in most routine clinical practices. Part of the objectives of this study was to investigate the utility of neuropathy screening tools that might be useful in poor-resource settings.

The BPNS has been used as a screening tool in HIV-SN [10], [11], [62], [67]. We found both the TCSS and UENS to be superior to the BPNS in the diagnosis of HIV-SN when using the triumvirate diagnostic criterion as a comparator. However, unlike the BPNS or TCSS, the UENS and triumvirate diagnostic criteria do not include a symptom assessment, which perhaps explains why the UENS produced the most similar results to the triumvirate. The UENS has been used in HIV-SN, and it correlated with measures of cutaneous autonomic function and pain [6].

### 4.5 Epidemiological studies

The population recruited to this deep profiling study had a prevalence of HIV-SN of 43%, with 75% of those reporting pain, which is similar to larger epidemiological studies [12]. In the cART era, HIV-SN prevalence is consistently reported at ∼40% [22], [49], [62], [67]. This supports the face validity of the triumvirate criteria used, as it is likely that these findings would be applicable to larger HIV populations and in different settings. However, this study did not find some of the well-established risk factors for HIV-SN of height, age, and sex in the cART era to be important. This study was powered as a deep phenotyping study and was not designed to be large enough to elucidate these risk factors compared to larger epidemiological studies [10], [22], [40], [62], [72].

A potential limitation of the study is that several items of the triumvirate definition for HIV-SN are compared and contrasted between the HIV–No SN group and HIV-SN group. It is clear from our results that no single parameter alone should be used in isolation for the diagnosis at the level of the individual patient. There is thus potential for a circular argument relating to the QST and IENFD findings. Therefore, we performed an exercise where we post hoc altered the case definition criteria to test whether these substantially changed the group allocations. Use of different combinations of the triumvirate shows that the neuropathy diagnosis of individual participants was not dramatically altered by removal of individual items from the triumvirate. Removal of QST resulted in 8 (12%) of the participants having an altered diagnosis, and IENFD only 6 (9%). Removal of SNE alters diagnosis in 17 (26%), demonstrating that a careful structured clinical examination is most critical in making the diagnosis of HIV-SN. There is an argument that only loss of function in the threshold QST parameters should be used in HIV-SN diagnosis. The percentages of participants in the HIV-SN group where such abnormal threshold values were present and used as part of the case definition are as follows: CDT (21%), WDT (14%), MDT (54%), VDT (50%), HPT (29%), and CPT (0%). When only loss-of-threshold QST measures in conjunction with the triumvirate definition are used, only in 2 individuals (7% of HIV-SN group) was this critical in the diagnosis, resulting in an altered group allocation from HIV-SN to HIV–No SN, demonstrating that this did not substantially change group allocations.

### 4.6 Summary

This study demonstrated that the most frequent changes seen in QST are loss of function in the large fibre parameters of mechanical and vibration detection, despite HIV-SN often being described as a SFN. The diagnostic limitation of QST, IENFD and clinical examination in isolation has also been demonstrated. This study has validated the use of UENS and TCSS in the diagnosis of HIV-SN, both of which were superior to the BPNS in the diagnosis of HIV-SN. This study also supported the growing evidence that lipid dyslipidaemia may play a role in the development of HIV-SN. Painful HIV-SN also appears to have a greater impact on quality of life compared to other pain syndromes.

Future work should determine the triumvirate diagnostic criteria’s validity and utility in other HIV populations. A detailed examination of the components of the triumvirate should also be undertaken, as there is an urgent need to produce a simple and robust diagnostic tool for large epidemiological studies in low-resource environments and for use in future therapeutic efficacy trials.

## Conflict of interest statement

DLHB has undertaken consultancy for Pfizer and Astellas. He is a principal investigator in the European Union-funded private–public partnership Innovative Medicines Initiative Joint Undertaking—EuroPain (www.imieuropain.org), and several pharmaceutical companies are also engaged in that project. EKK has been a member of IMI EuroPain; she was also supported by intramural funding from the Ruhr University Bochum (FoRUMl grant K046-10) and has received a travel grant from Mundipharma as well as speaker fees from Grunenthal. ASCR undertakes consulting and advisory board work for Imperial College Consultants and in the past 36 months has received fees from Spinifex Pharmaceuticals, Astellas, Servier, Allergan, Asahi Kasei, and Medivir; has share options in Spinifex Pharmaceuticals; has received consultancy fees from the Wellcome Trust Seeding Drug Discovery Committee; and is a principal investigator in the European Union–funded private–public partnership Innovative Medicines Initiative Joint Undertaking—EuroPain (www.imieuropain.org), and several pharmaceutical companies are also engaged in that project. Through EuroPain, ASCR’s laboratory has received funding from Pfizer and Astellas. The other authors report no conflict of interest.

## Appendix A Supplementary data

Supplemental Document 1

Supplemental Document 2 DFNS QST Equipment used, Heat Suprathreshold protocol and Healthy Control Inclusion and Exclusion criteria.

Supplemental Document 3 The structured neurological examination of the upper and lower limb was designed by an experienced neurologist (DB), to exam in detail, clinical neurological signs which might be present in a peripheral neuropathy.

Supplemental Document 4 Mean values of QST parameters after z-score transformation for the three groups. Dynamic mechanical allodynia (DMA) and paradoxical heat sensations (PHS) are presented as mean number of events. _∗_z-score transformed values outside 95% CI range of the DFNS normal values, percentages in brackets. CDT = Cold Detection Threshold, WDT = Warm Detection Threshold, TSL = Thermal Sensory Limen, CPT = Cold Pain Threshold, HPT = Heat Pain Threshold, MPT = Mechanical Pain Threshold, MPS = Mechanical Pain Sensitivity, WUR = Wind-Up Ratio, MDT = Mechanical Detection Threshold, VDT = Vibration Detection Threshold.

Supplemental Document 5 Correlation of IENFD and individual thermal and mechanical QST parameters. Pearson’s correlation *r* values. IENFD = Intra Epidermal Fibre Density, QST = Quantitative Sensory Testing, CDT = Cold Detection Threshold, WDT = Warm Detection Threshold, TSL = Thermal Sensory Limen, CPT = Cold Pain Threshold, HPT = Heat Pain Threshold, MPT = Mechanical Pain Threshold, MPS = Mechanical Pain Sensitivity, WUR = Wind-Up Ratio, MDT = Mechanical Detection Threshold, VDT = Vibration Detection Threshold.

Supplemental Document 6 Mean and SD scores for NPSI pain descriptors for patients with painful HIV-SN (*n* = 19). NPSI = Neuropathic Pain Symptom Inventory, HIV-SN = HIV Sensory Neuropathy.

Supplemental Document 7 Psychological and Sleep instruments questionnaire returned and completed per group. Numbers completed with percentages in brackets. HIV-SN = HIV sensory neuropathy, HIV–No SN = No HIV Sensory Neuropathy, ISI = Insomnia Severity Index, DAPOS = Depression Anxiety Positive Outlook Scale, PASS-20 = Pain Anxiety Symptom Scale Short Form, PCS = Pain Catastophizing Scale, SF-36 = Short Form (36) Health Survey.
