# Supplementary material for: Sensory, psychological, and metabolic dysfunction in HIV-associated peripheral neuropathy: A cross-sectional deep profiling study
Source: Pain. 2014 Sep;155(9):1846–60. doi: 10.1016/j.pain.2014.06.014 (PMC4165602; doi:10.1016/j.pain.2014.06.014)
Supplement: Supplemental Document 1 [file mmc1.doc]

| DFNS Identifier | |  | | | | | | | | | | |  | | | | | |  | |  |
| --- | --- | --- | --- | --- | --- | --- | --- | --- | --- | --- | --- | --- | --- | --- | --- | --- | --- | --- | --- | --- | --- |
| PINS-HIV No | |  | | | | | | | | | | |  | | | | | |  | |  |
| Hospital Number | |  | | | | | | | | | | |  | | | | | |  | |  |
|  | |  | | | | | | | | | | |  | | | | | |  | | |
| Dates of interview/examination | |  | | | | | | | | | | |  | | | | | | | | |
|  | |  | | | | | | | | | | |  | | | | | | | | |
| Date of Birth | |  | | | |  | | | | | | |  | | | | | |  | |  |
| Chromosomal Gender | | M/F | | | |  | | | | | | |  | | | | | |  | |  |
| Height | | m | | | |  | | | | | | |  | | | | | |  | |  |
| Weight | | kg | | | |  | | | | | | |  | | | | | |  | |  |
| Waist circumference | | cm | | | |  | | | | | | |  | | | | | |  | |  |
| Hip circumference | | cm | | | |  | | | | | | |  | | | | | |  | |  |
|  | |  | | | | | | | | | | |  | | | | | |  | |  |
| Ethnicity* | |  | | | | | | | | | | |  | | | | | |  | |  |
| Maternal Grandmother birthplace | |  | | | | | | | | | | |  | | | | | |  | |  |
| Maternal Grandfather birthplace | |  | | | | | | | | | | |  | | | | | |  | |  |
| Paternal Grandmother birthplace | |  | | | | | | | | | | |  | | | | | |  | |  |
| Paternal Grandfather birthplace | |  | | | | | | | | | | |  | | | | | |  | |  |
|  | |  | | | | | | | | | | |  | | | | | |  | |  |
| Smoking | | Y/N | | | | | | | | | | |  | | | | | | No/week | |  |
| Previously? | | Y/N | | | | | | | | | | |  | | | | | | No/week | |  |
| Date Quit | |  | | | | | | | | | | |  | | | | | | | |  |
|  | |  | | | | | | | | | | |  | | | | | | | | |
| Alcohol | | Y/N | | | | | | | | | | |  | | | | | | | |  |
| Previously? | |  | | | | | | | | | | |  | | | | | | |  | |
| Date Quit | |  | | | | | | | | | | |  | | | | | | |  | |
|  | | | | | | | | | | | | | | | | | | | |  | |
|  | Drink Type | | | | | | | | | Freq code | | | | Quant | | U | | | |  | |
|  | Beer/Larger/Stout/Cider/Alcopop | | | | | | | | |  | | | |  | |  | | | |  |  |
|  | Shandy | | | | | | | | |  | | | |  | |  | | | |  |  |
|  | Spirits: Gin/Whiskey/Rum/Vodka/Brady/Cocktail | | | | | | | | |  | | | |  | |  | | | |  |  |
|  | Wine Champagne | | | | | | | | |  | | | |  | |  | | | |  |  |
|  | Fortified Wine | | | | | | | | |  | | | |  | |  | | | |  |  |
|  | Total | | | | | | | | | | | | | | |  | | | | U/week |  |
|  |  |  |  |  |  |  |  |  |  |  |  |  |  |  |  |  |  |  |  |  |  |
| Date of HIV Diagnosis | | | | | | | | | |  | | | |  | | | | | |  |  |
| Date of AV treatment start | | | | | | | | | |  | | | |  | | | | | |  |  |
|  |  | | | | | | | | | | | | |  | | | | | |  |  |
|  |  | | | | | | | | 1st | | 2nd | | | | | | | | |  |  |
| Lying-Standing BP | Lying | | | | | | | |  | |  | | | | | | | | |  |  |
|  | Standing | | | | | | | |  | |  | | | | | | | | |  |  |
|  |  | | | | | | | | | | | | |  | | | | | |  |  |
|  | Diagnosis | | | | | | | | | | | | | Date of Diagnosis | | | | | |  |  |
| Other Past Medical History | 1. | | | | | | | | | | | | |  | | | | | |  |  |
|  | 2. | | | | | | | | | | | | |  | | | | | |  |  |
|  | 3. | | | | | | | | | | | | |  | | | | | |  |  |
|  | 4. | | | | | | | | | | | | |  | | | | | |  |  |
|  | 5. | | | | | | | | | | | | |  | | | | | |  |  |
|  | 6. | | | | | | | | | | | | |  | | | | | |  |  |
|  | 7. | | | | | | | | | | | | |  | | | | | |  |  |
|  | 8. | | | | | | | | | | | | |  | | | | | |  |  |
|  | 9. | | | | | | | | | | | | |  | | | | | |  |  |
|  | 10. | | | | | | | | | | | | |  | | | | | |  |  |
|  | 11. | | | | | | | | | | | | |  | | | | | |  |  |
|  | 12. | | | | | | | | | | | | |  | | | | | |  |  |
|  | 13. | | | | | | | | | | | | |  | | | | | |  |  |
|  | 14. | | | | | | | | | | | | |  | | | | | |  |  |
|  | 15. | | | | | | | | | | | | |  | | | | | |  |  |
|  | 16. | | | | | | | | | | | | |  | | | | | |  |  |
|  | 17. | | | | | | | | | | | | |  | | | | | |  |  |
|  | 18. | | | | | | | | | | | | |  | | | | | |  |  |
|  | 19. | | | | | | | | | | | | |  | | | | | |  |  |
|  | 20. | | | | | | | | | | | | |  | | | | | |  |  |
|  |  | | | | | | | | | | |  | | | | | | | |  |  |
| Current AV drugs | | | | | | | | | | Dose/day | | Date Commenced | | | | | | | |  |  |
| 1. | | | | | | | | | |  | |  | | | | | | | |  |  |
| 2. | | | | | | | | | |  | |  | | | | | | | |  |  |
| 3. | | | | | | | | | |  | |  | | | | | | | |  |  |
| 4. | | | | | | | | | |  | |  | | | | | | | |  |  |
| 5. | | | | | | | | | |  | |  | | | | | | | |  |  |
|  |  | | | | | | | | | | |  | | | | | | | |  |  |
| Past AV drugs | | | | | | | | | | Dose/day | | Date Commenced | | | | | | | | Date Finished |  |
| 1. | | | | | | | | | |  | |  | | | | | | | |  |  |
| 2. | | | | | | | | | |  | |  | | | | | | | |  |  |
| 3. | | | | | | | | | |  | |  | | | | | | | |  |  |
| 4. | | | | | | | | | |  | |  | | | | | | | |  |  |
| 5. | | | | | | | | | |  | |  | | | | | | | |  |  |
| 6. | | | | | | | | | |  | |  | | | | | | | |  |  |
| 7. | | | | | | | | | |  | |  | | | | | | | |  |  |
| 8. | | | | | | | | | |  | |  | | | | | | | |  |  |
| 9. | | | | | | | | | |  | |  | | | | | | | |  |  |
| 10. | | | | | | | | | |  | |  | | | | | | | |  |  |
|  |  | | | | | | | | | | |  | | | | | | | |  |  |
|  |  | | | | | | | | | | |  | | | | | | | |  |  |
| Current Drug treatment | | | | | | | | | | Dose/day | | Date Commenced | | | | | | | |  |  |
| 1. | | | | | | | | | |  | |  | | | | | | | |  |  |
| 2. | | | | | | | | | |  | |  | | | | | | | |  |  |
| 3. | | | | | | | | | |  | |  | | | | | | | |  |  |
| 4. | | | | | | | | | |  | |  | | | | | | | |  |  |
| 5. | | | | | | | | | |  | |  | | | | | | | |  |  |
| 6. | | | | | | | | | |  | |  | | | | | | | |  |  |
| 7. | | | | | | | | | |  | |  | | | | | | | |  |  |
| 8 | | | | | | | | | |  | |  | | | | | | | |  |  |
| 9. | | | | | | | | | |  | |  | | | | | | | |  |  |
| 10. | | | | | | | | | |  | |  | | | | | | | |  |  |
| 11. | | | | | | | | | |  | |  | | | | | | | |  |  |
| 12. | | | | | | | | | |  | |  | | | | | | | |  |  |
| 13. | | | | | | | | | |  | |  | | | | | | | |  |  |
| 14. | | | | | | | | | |  | |  | | | | | | | |  |  |
| 15. | | | | | | | | | |  | |  | | | | | | | |  |  |
| 16. | | | | | | | | | |  | |  | | | | | | | |  |  |
| 17. | | | | | | | | | |  | |  | | | | | | | |  |  |
| 18. | | | | | | | | | |  | |  | | | | | | | |  |  |
| 19. | | | | | | | | | |  | |  | | | | | | | |  |  |
| 20. | | | | | | | | | |  | |  | | | | | | | |  |  |
|  |  | | | | | | | | |  | |  | | | | | | | |  |  |
| Previous Drug treatment | | | | | | | | | | Dose/day | | Date Commenced | | | | | | | | Date Finished |  |
| 1. | | | | | | | | | |  | |  | | | | | | | |  |  |
| 2. | | | | | | | | | |  | |  | | | | | | | |  |  |
| 3. | | | | | | | | | |  | |  | | | | | | | |  |  |
| 4. | | | | | | | | | |  | |  | | | | | | | |  |  |
| 5. | | | | | | | | | |  | |  | | | | | | | |  |  |
| 6. | | | | | | | | | |  | |  | | | | | | | |  |  |
| 7. | | | | | | | | | |  | |  | | | | | | | |  |  |
| 8 | | | | | | | | | |  | |  | | | | | | | |  |  |
| 9. | | | | | | | | | |  | |  | | | | | | | |  |  |
| 10. | | | | | | | | | |  | |  | | | | | | | |  |  |
| 11. | | | | | | | | | |  | |  | | | | | | | |  |  |
| 12. | | | | | | | | | |  | |  | | | | | | | |  |  |
| 13. | | | | | | | | | |  | |  | | | | | | | |  |  |
| 14. | | | | | | | | | |  | |  | | | | | | | |  |  |
| 15. | | | | | | | | | |  | |  | | | | | | | |  |  |
| 16. | | | | | | | | | |  | |  | | | | | | | |  |  |
| 17. | | | | | | | | | |  | |  | | | | | | | |  |  |
| 18. | | | | | | | | | |  | |  | | | | | | | |  |  |
| 19. | | | | | | | | | |  | |  | | | | | | | |  |  |
| 20. | | | | | | | | | |  | |  | | | | | | | |  |  |
|  |  | | | | | | | | | | |  | | | | | | | | |  |
|  |  |  |  |  |  |  |  |  |  |  |  |  |  |  |  |  |  |  |  |  |  |
| Neuropathy Symptoms? | | |  | | | | | | | Date of onset | | Comments | | | | | | | | |  |
| Paraesthesia | | | Y/N | | | | | | |  | |  | | | | | | | | |  |
| Numbness | | | Y/N | | | | | | |  | |  | | | | | | | | |  |
| Weakness | | | Y/N | | | | | | |  | |  | | | | | | | | |  |
| Postural hypotension | | | Y/N | | | | | | |  | |  | | | | | | | | |  |
| Urinary dysfunction | | | Y/N | | | | | | |  | |  | | | | | | | | |  |
| Erectile dysfunction | | | Y/N | | | | | | |  | |  | | | | | | | | |  |
| Nocturnal diarrhoea | | | Y/N | | | | | | |  | |  | | | | | | | | |  |
| Pain | | | Y/N | | | | | | |  | |  | | | | | | | | |  |
|  | |  | | | | | | | | | |  | | | | | | | |  |  |
| Family history of neuropathy? | | Family member | | | | | | | | | | Diagnosis | | | | | | | | |  |
| Y/N | | 1. | | | | | | | | | |  | | | | | | | | |  |
|  | | 2. | | | | | | | | | |  | | | | | | | | |  |
|  | | 3. | | | | | | | | | |  | | | | | | | | |  |
|  | | 4. | | | | | | | | | |  | | | | | | | | |  |
|  | |  | | | | | | | | | |  | | | | | | | |  |  |
| Please rate the severity of each of the following symptoms on a scale of 1(mild) to 10 (severe): | | (i) Pain, aching, burning in feet, legs (BPNS/TCSS) | | | | | | | | | |  | | | | | | | | 0-10 or Always been normal (AN) |  |
|  | | (ii) “Pins and needles” in feet, legs (BPNS) | | | | | | | | | |  | | | | | | | | 0-10 or Always been normal (AN) |  |
|  | | (iii) Numbness (lack of feeling) in feet, legs. (BPNS/TCSS) | | | | | | | | | |  | | | | | | | | 0-10 or Always been normal (AN) |  |
|  | | (iv) Tingling in feet (TCSS) | | | | | | | | | | Y/N | | | | | | | |  |  |
|  | | (v) Weakness in feet (TCSS) | | | | | | | | | | Y/N | | | | | | | |  |  |
|  | |  | | | | | | | | | |  | | | | | | | |  |  |
|  | |  | | | | | | | | | |  | | | | | | | |  |  |
|  | |  | | | | | | | | | |  | | | | | | | |  |  |
|  | |  | | | | | | | | | |  | | | | | | | |  |  |
| Physician’s Neuropathy Differential Diagnosis | | | | | | | | | | | |  | | | | | | | |  |  |
| 1. | | | | | | | | | | | |  | | | | | | | |  |  |
| 2. | | | | | | | | | | | |  | | | | | | | |  |  |
| 3. | | | | | | | | | | | |  | | | | | | | |  |  |
| 4. | | | | | | | | | | | |  | | | | | | | |  |  |
| 5. | | | | | | | | | | | |  | | | | | | | |  |  |
|  | |  | | | | | | | | | |  | | | | | | | |  |  |
|  | |  | | | | | | | | | |  | | | | | | | |  |  |
|  | |  | | | | | | | | | |  | | | | | | | |  |  |
|  | |  | | | | | | | | | |  | | | | | | | |  |  |
|  | |  | | | | | | | | | |  | | | | | | | |  |  |
|  | |  | | | | | | | | | |  | | | | | | | |  |  |
|  | |  | | | | | | | | | |  | | | | | | | |  |  |
|  | |  | | | | | | | | | |  | | | | | | | |  |  |
| **Medical Notes:** | |  | | | | | | | | | |  | | | | | | | |  |  |
|  | |  | | | | | | | | | | Value | | | | | Date of test | | | |  |
| HIV | | Current CD4+ count (450-1660) | | | | | | | | | |  | | | | |  | | | |  |
|  | | Current CD4+ % (30-65%) | | | | | | | | | |  | | | | |  | | | |  |
|  | | Nadir CD4+ count (450-1660) | | | | | | | | | |  | | | | |  | | | |  |
|  | | Nadir CD4+ % (30-65%) | | | | | | | | | |  | | | | |  | | | |  |
|  | | Current HIV-1 RNA (viral load RNA/ml) | | | | | | | | | |  | | | | |  | | | |  |
|  | | Peak HIV-1 RNA (viral load RNA/ml) | | | | | | | | | |  | | | | |  | | | |  |
|  | |  | | | | | | | | | |  | | | | |  | | | |  |
|  | |  | | | | | | | | | | Concentration | | | | | Date of test | | | |  |
| Thyroid function | | T4 _free_ (9.0-26.0 pmol/l) | | | | | | | | | |  | | | | |  | | | |  |
|  | | TSH (0.3-4.2mU/l) | | | | | | | | | |  | | | | |  | | | |  |
|  | | T3 | | | | | | | | | |  | | | | |  | | | |  |
|  | |  | | | | | | | | | |  | | | | | |  | | |  |
| Lipids | | Cholesterol (<5.0 mmol/l) | | | | | | | | | |  | | | | |  | | | |  |
|  | | Triglycerides (0-2.0 mmol/l) | | | | | | | | | |  | | | | |  | | | |  |
|  | | HDL (0.9-1.9 mmol/l) | | | | | | | | | |  | | | | |  | | | |  |
|  | | LDL (2.0-5.0 mmol/l) | | | | | | | | | |  | | | | |  | | | |  |
|  | | HDL: Cholesterol ratio (0-5.0) | | | | | | | | | |  | | | | |  | | | |  |
|  | | | | | | | | | | | |  | | | | | | | | |  |
|  | | | | | | | | | | | | Value | | | | | Date of test | | | |  |
| FBC | Hb | | | | | | | | | | |  | | | | |  | | | |  |
|  | WCC | | | | | | | | | | |  | | | | |  | | | |  |
|  | Plts | | | | | | | | | | |  | | | | |  | | | |  |
|  |  | | | | | | | | | | |  | | | | |  | | | |  |
|  |  | | | | | | | | | | | Value | | | | | Date of test | | | |  |
| Blood electrolytes | Na^+^ | | | | | | | | | | |  | | | | |  | | | |  |
|  | K^+^ | | | | | | | | | | |  | | | | |  | | | |  |
|  | Ur | | | | | | | | | | |  | | | | |  | | | |  |
|  | Cr | | | | | | | | | | |  | | | | |  | | | |  |
|  | | | | | | | | | | | |  | | | | | | | |  |  |
|  |  |  |  |  |  |  |  |  |  |  |  | Value | | | | | Date of test | | | |  |
| Liver Function Enzymes | Bilirubin | | | | | | | | | | |  | | | | |  | | | |  |
|  | Alanine Transaminase | | | | | | | | | | |  | | | | |  | | | |  |
|  | Alkaline Phosphatase | | | | | | | | | | |  | | | | |  | | | |  |
|  | Gamma-Glutamyl Transpeptidase | | | | | | | | | | |  | | | | |  | | | |  |
|  | | | | | | | | | | | |  | | | | | | | |  |  |
|  |  |  |  |  |  |  |  |  |  |  |  | Value | | | | | Date of test | | | |  |
| Blood Glucose | Random | | | | | | | | | | |  | | | | |  | | | |  |
|  | Hb_A1C_ | | | | | | | | | | |  | | | | |  | | | |  |
|  |  | | | | | | | | | | |  | | | | | | | |  |  |
|  |  | | | | | | | | | | |  | | | | | | | |  |  |
|  |  | | | | | | | | | | |  | | | | | | | |  |  |
|  |  | | | | | | | | | | |  | | | | | | | |  |  |
|  |  | | | | | | | | | | |  | | | | | | | |  |  |
|  |  | | | | | | |  | | | |  | | | | | | | |  |  |
|  |  | | | | | | | Test | | | | Value | | | | | | | | Date of test |  |
|  | Vitamin B_12_ (160-800nq/l) | | | | | | | Y/N | | | |  | | | | | | | |  |  |
|  | Syphilis VDRL | | | | | | | Y/N | | | | Neg/Pos | | | | | | | |  |  |
|  | Syphilis TPPA | | | | | | | Y/N | | | | Neg/Pos | | | | | | | |  |  |
|  |  | | | | | | | | | | |  | | | | | | | |  |  |
|  | Hepatitis B core antigen  Hepatitis C RNA | | | | | | | | | | | Y/N | | | | | | | | Neg/Pos |  |
|  |  |  |  |  |  |  |  |  |  |  |  | Y/N | | | Neg/Pos | | | | |  |  |
|  | Vitamin B_12_ (160-800nq/l) | | | | | | | | | | | Y/N | | |  | | | | |  |  |
|  | Syphilis VDRL | | | | | | | | | | | Y/N | | | Neg/Pos | | | | |  |  |
|  | Syphilis TPPA | | | | | | | | | | | Y/N | | | Neg/Pos | | | | |  |  |
|  | Hepatitis B core antigen | | | | | | | | | | | Y/N | | | Neg/Pos | | | | |  |  |
|  | Hepatitis C RNA | | | | | | | | | | | Y/N | | | Neg/Pos | | | | |  |  |
|  |  | | | | | | | | | | |  | | | | | | | |  |  |
|  | | | | | | | | | | | | | | | | | | | |  |  |
| Neurophysiology |  | | | | | | | | | | | | | |  |  |  |  | | --- | --- | --- | --- | | | | | | |  |  |
|  | Median n. | | | | | | | | | | | | | |  |  |  |  | | --- | --- | --- | --- | | | | | | |  |  |
|  | Ulnar n. | | | | | | | | | | | | | |  |  |  |  | | --- | --- | --- | --- | | | | | | |  |  |
|  | Tibial n. | | | | | | | | | | | | | |  |  |  |  | | --- | --- | --- | --- | | | | | | |  |  |
|  | C. Peroneal n. | | | | | | | | | | | | | |  |  |  |  | | --- | --- | --- | --- | | | | | | |  |  |
|  |  | | | | | | | | | | | | |  | | | | | |  |  |
|  |  | | | | | | | | | | | | |  | | | | | |  |  |
|  |  | | | | | | | | | | | | |  | | | | | |  |  |
|  |  | | | | | | | | | | | | |  | | | | | |  |  |
|  |  | | | | | | | | | | | | |  | | | | | |  |  |
|  |  | | | | | | | | | | | | |  | | | | | |  |  |
|  |  | | | | | | | | | | | | |  | | | | | |  |  |
|  |  | | | | | | | | | | | | |  | | | | | |  |  |
|  |  | | | | | | | | | | | | |  | | | | | |  |  |
|  |  | | | | | | | | | | | | |  | | | | | |  |  |
|  |  | | | | | | | | | | | | |  | | | | | |  |  |
|  |  | | | | | | | | | | | | |  | | | | | |  |  |
|  |  | | | | | | | | | | | | |  | | | | | |  |  |
|  |  | | | | | | | | | | | | |  | | | | | |  |  |
|  |  | | | | | | | | | | | | |  | | | | | |  |  |
|  |  | | | | | | | | | | | | |  | | | | | |  |  |
|  |  | | | | | | | | | | | | |  | | | | | |  |  |
|  |  | | | | | | | | | | | | |  | | | | | |  |  |
|  |  | | | | | | | | | | | | |  | | | | | |  |  |
|  |  | | | | | | | | | | | | |  | | | | | |  |  |
|  |  | | | | | | | | | | | | |  | | | | | |  |  |
|  |  | | | | | | | | | | | | |  | | | | | |  |  |
|  |  | | | | | | | | | | | | |  | | | | | |  |  |
|  |  | | | | | | | | | | | | |  | | | | | |  |  |
|  |  | | | | | | | | | | | | |  | | | | | |  |  |
|  |  | | | | | | | | | | | | |  | | | | | |  |  |
| **Questionnaires:** |  | | | | | | | | | | | | |  | | | | | |  |  |
| **Questionnaire 1**  7-day Pain Diary |  | | | | | | | | | | | | | Completed Questionnaire? | | | | | | Y/N |  |
|  |  | | | | | | | | | | | | | Completed Body map? | | | | | | Y/N |  |
|  | Score | | | | | | | | | | | | |  | | | | | |  |  |
| Day 1 am |  | | | | | | | | | | | | | 0-10 | | | | | |  |  |
| Day 1 pm |  | | | | | | | | | | | | | 0-10 | | | | | |  |  |
| Day 2 am |  | | | | | | | | | | | | | 0-10 | | | | | |  |  |
| Day 2 pm |  | | | | | | | | | | | | | 0-10 | | | | | |  |  |
| Day 3 am |  | | | | | | | | | | | | | 0-10 | | | | | |  |  |
| Day 3 pm |  | | | | | | | | | | | | | 0-10 | | | | | |  |  |
| Day 4 am |  | | | | | | | | | | | | | 0-10 | | | | | | Total |  |
| Day 4 pm |  | | | | | | | | | | | | | 0-10 | | | | | |  |  |
| Day 5 am |  | | | | | | | | | | | | | 0-10 | | | | | |  |  |
| Day 5 pm |  | | | | | | | | | | | | | 0-10 | | | | | |  |  |
| Day 6 am |  | | | | | | | | | | | | | 0-10 | | | | | |  |  |
| Day 6 pm |  | | | | | | | | | | | | | 0-10 | | | | | |  |  |
| Day 7 am |  | | | | | | | | | | | | | 0-10 | | | | | |  |  |
| Day 7 pm |  | | | | | | | | | | | | | 0-10 | | | | | |  |  |
|  |  | | | | | | | | | | | | |  | | | | | |  |  |
| **Questionnaire 2** Neuropathic Pain Symptom Inventory (NPSI) |  | | | | | | | | | | | | | Completed Questionnaire? | | | | | | Y/N |  |
|  | Score | | | | | | | | | | | | |  | | | | | |  |  |
| Q1 Burning |  | | | | | | | | | | | | | 0-10 | | | | | |  |  |
| Q2 Squeezing |  | | | | | | | | | | | | | 0-10 | | | | | |  |  |
| Q3 Pressure |  | | | | | | | | | | | | | 0-10 | | | | | |  |  |
| Q4 Degree of Spontaneous ongoing pain |  | | | | | | | | | | | | | 0-5 | | | | | |  |  |
| Q5 Electric shocks |  | | | | | | | | | | | | | 0-10 | | | | | | Total |  |
| Q6 Stabbing |  | | | | | | | | | | | | | 0-10 | | | | | |  |  |
| Q7 Degree of spontaneous paroxysmal pain |  | | | | | | | | | | | | | 0-5 | | | | | |  |  |
| Q8 Brush-evoked |  | | | | | | | | | | | | | 0-10 | | | | | |  |  |
| Q9 Pressure evoked |  | | | | | | | | | | | | | 0-10 | | | | | |  |  |
| Q10 Cold-evoked |  | | | | | | | | | | | | | 0-10 | | | | | |  |  |
| Q11 Pins and Needles |  | | | | | | | | | | | | | 0-10 | | | | | |  |  |
| Q12 Tingling |  | | | | | | | | | | | | | 0-10 | | | | | |  |  |
|  | | | | | | | | | | | |  | | | | | | | | |  |
|  | | | | | | | | | | | | | | | | | | | | |  |
|  | | | | | | | | | | | | | | | | | | | | |  |
|  | | | | | | | | | | | | | | | | | | | | |  |
| **Questionnaire 3:**  Depression Anxiety Positive Outlook Scale (DAPOS) | | | | | | | | | | | | | | Completed Questionnaire? | | | | | | Y/N |  |
|  | Score | | | | | | | | | | | | |  | | | | | |  |  |
| Q1 |  | | | | | | | | | | | | | 0-5 | | | | | |  |  |
| Q2 |  | | | | | | | | | | | | | 0-5 | | | | | |  |  |
| Q3 |  | | | | | | | | | | | | | 0-5 | | | | | |  |  |
| Q4 |  | | | | | | | | | | | | | 0-5 | | | | | |  |  |
| Q5 |  | | | | | | | | | | | | | 0-5 | | | | | | Total |  |
| Q6 |  | | | | | | | | | | | | | 0-5 | | | | | |  |  |
| Q7 |  | | | | | | | | | | | | | 0-5 | | | | | |  |  |
| Q8 |  | | | | | | | | | | | | | 0-5 | | | | | |  |  |
| Q9 |  | | | | | | | | | | | | | 0-5 | | | | | |  |  |
| Q10 |  | | | | | | | | | | | | | 0-5 | | | | | |  |  |
|  |  | | | | | | | | | | | | |  | | | | | |  |  |
| **Questionnaire 4:**  Pain Catastrophising Scale (PCS) | | | | | | | | | | | | | | Completed Questionnaire? | | | | | | Y/N |  |
|  | Score | | | | | | | | | | | | |  | | | | | |  |  |
| Q1 |  | | | | | | | | | | | | | 0-4 | | | | | |  |  |
| Q2 |  | | | | | | | | | | | | | 0-4 | | | | | |  |  |
| Q3 |  | | | | | | | | | | | | | 0-4 | | | | | |  |  |
| Q4 |  | | | | | | | | | | | | | 0-4 | | | | | |  |  |
| Q5 |  | | | | | | | | | | | | | 0-4 | | | | | |  |  |
| Q6 |  | | | | | | | | | | | | | 0-4 | | | | | | Total |  |
| Q7 |  | | | | | | | | | | | | | 0-4 | | | | | |  |  |
| Q8 |  | | | | | | | | | | | | | 0-4 | | | | | |  |  |
| Q9 |  | | | | | | | | | | | | | 0-4 | | | | | |  |  |
| Q10 |  | | | | | | | | | | | | | 0-4 | | | | | |  |  |
| Q11 |  | | | | | | | | | | | | | 0-4 | | | | | |  |  |
| Q12 |  | | | | | | | | | | | | | 0-4 | | | | | |  |  |
| Q13 |  | | | | | | | | | | | | | 0-4 | | | | | |  |  |
|  |  | | | | | | | | | | | | |  | | | | | |  |  |
| **Questionnaire 5.**  Short-form Pain Anxiety Symptoms Scale (PASS-20) | | | | | | | | | | | | | | Completed Questionnaire? | | | | | | Y/N |  |
|  | Score | | | | | | | | | | | | |  | | | | | |  |  |
| Q1 |  | | | | | | | | | | | | | 0-5 | | | | | |  |  |
| Q2 |  | | | | | | | | | | | | | 0-5 | | | | | |  |  |
| Q3 |  | | | | | | | | | | | | | 0-5 | | | | | |  |  |
| Q4 |  | | | | | | | | | | | | | 0-5 | | | | | |  |  |
| Q5 |  | | | | | | | | | | | | | 0-5 | | | | | |  |  |
| Q6 |  | | | | | | | | | | | | | 0-5 | | | | | |  |  |
| Q7 |  | | | | | | | | | | | | | 0-5 | | | | | |  |  |
| Q8 |  | | | | | | | | | | | | | 0-5 | | | | | | Total |  |
| Q9 |  | | | | | | | | | | | | | 0-5 | | | | | |  |  |
| Q10 |  | | | | | | | | | | | | | 0-5 | | | | | |  |  |
| Q11 |  | | | | | | | | | | | | | 0-5 | | | | | |  |  |
| Q12 |  | | | | | | | | | | | | | 0-5 | | | | | |  |  |
| Q13 |  | | | | | | | | | | | | | 0-5 | | | | | |  |  |
| Q14 |  | | | | | | | | | | | | | 0-5 | | | | | |  |  |
| Q15 |  | | | | | | | | | | | | | 0-5 | | | | | |  |  |
| Q16 |  | | | | | | | | | | | | | 0-5 | | | | | |  |  |
| Q17 |  | | | | | | | | | | | | | 0-5 | | | | | |  |  |
| Q18 |  | | | | | | | | | | | | | 0-5 | | | | | |  |  |
| Q19 |  | | | | | | | | | | | | | 0-5 | | | | | |  |  |
| Q20 |  | | | | | | | | | | | | | 0-5 | | | | | |  |  |
|  |  | | | | | | | | | | | | |  | | | | | |  |  |
| **Questionnaire 6:**  Insomnia Severity Index (ISI) | | | | | | | | | | | | | | Completed Questionnaire? | | | | | | Y/N |  |
|  | Score | | | | | | | | | | | | |  | | | | | |  |  |
| Q1a Initial |  | | | | | | | | | | | | | 0-4 | | | | | |  |  |
| Q1bMiddle |  | | | | | | | | | | | | | 0-4 | | | | | |  |  |
| Q1cTerminal |  | | | | | | | | | | | | | 0-4 | | | | | | Total |  |
| Q2 Satisfaction |  | | | | | | | | | | | | | 0-4 | | | | | |  |  |
| Q3 Interference |  | | | | | | | | | | | | | 0-4 | | | | | |  |  |
| Q4 Noticeability |  | | | | | | | | | | | | | 0-4 | | | | | |  |  |
| Q5 Distress |  | | | | | | | | | | | | | 0-4 | | | | | |  |  |
|  |  | | | | | | | | | | | | |  | | | | | |  |  |
| **Questionnaire7:**  Brief Pain Inventory – 7-item pain interference subscore. | | | | | | | | | | | | | | Completed Questionnaire? | | | | | | Y/N |  |
|  | Score | | | | | | | | | | | | |  | | | | | |  |  |
| Q1 General activities |  | | | | | | | | | | | | | 0-10 | | | | | |  |  |
| Q2 Mood |  | | | | | | | | | | | | | 0-10 | | | | | |  |  |
| Q3 Walking |  | | | | | | | | | | | | | 0-10 | | | | | | Total |  |
| Q4 Work |  | | | | | | | | | | | | | 0-10 | | | | | |  |  |
| Q5 Relationships |  | | | | | | | | | | | | | 0-10 | | | | | |  |  |
| Q6 Sleep |  | | | | | | | | | | | | | 0-10 | | | | | |  |  |
| Q7 Enjoyment of Life |  | | | | | | | | | | | | | 0-10 | | | | | |  |  |
|  |  | | | | | | | | | | | | |  | | | | | |  |  |
| **Questionnaire 8:**  SF-36 |  | | | | | | | | | | | | | Completed Questionnaire? | | | | | | Y/N |  |
|  | Sub-Scores | | | | | | | | | | | | |  | | | | | |  |  |
| SS1 Physical Functioning (PF) |  | | | | | | | | | | | | |  | | | | | |  |  |
| SS2 Role Limitations due to Physical Health (RP) |  | | | | | | | | | | | | |  | | | | | |  |  |
| SS3 Bodily Pain (BP) |  | | | | | | | | | | | | |  | | | | | |  |  |
| SS4 General Health (BP) |  | | | | | | | | | | | | |  | | | | | |  |  |
| SS5 Vitality (VT) |  | | | | | | | | | | | | |  | | | | | |  |  |
| SS6 Social Functioning (GH) |  | | | | | | | | | | | | |  | | | | | |  |  |
| SS7 Role Limitations due to Emotional problems (VT) |  | | | | | | | | | | | | |  | | | | | |  |  |
| SS8 Mental Health |  | | | | | | | | | | | | |  | | | | | |  |  |
|  |  | | | | | | | | | | | | |  | | | | | |  |  |
|  |  | | | | | | | | | | | | | Date | | | | | |  |  |
| Blood taken? | Y/N | | | | | | | | | | | | |  | | | | | |  |  |
| Skin punch Bx taken? | Y/N | | | | | | | | | | | | |  | | | | | |  |  |
|  |  | | | | | | | | | | | | | | | | | | | |  |
| IENFD result |  | | | | | | | | | | | |  | | | | | | |  | |
|  |  | | | | | |  | | | | | | | | | | | | |  | |
| QST Data | Left | | |  | Right | |  | | | | | | | | | | | | |  | |
| CDT |  | | |  |  | | ^0^C | | | | | | | | | | | | |  | |
| WDT |  | | |  |  | | ^0^C | | | | | | | | | | | | |  | |
| TSL |  | | |  |  | | ^0^C | | | | | | | | | | | | |  | |
| PHS |  | | |  |  | |  | | | | | | | | | | | | |  | |
| CPT |  | | |  |  | | ^0^C | | | | | | | | | | | | |  | |
| HPT |  | | |  |  | | ^0^C | | | | | | | | | | | | |  | |
| MDT |  | | |  |  | | mN | | | | | | | | | | | | |  | |
| MPT |  | | |  |  | | mN | | | | | | | | | | | | |  | |
| MPS |  | | |  |  | | log | | | | | | | | | | | | |  | |
| DMA |  | | |  |  | | log | | | | | | | | | | | | |  | |
| WUR |  | | |  |  | |  | | | | | | | | | | | | |  | |
| VDT |  | | |  |  | | /8 | | | | | | | | | | | | |  | |
| PPT |  | | |  |  | | kPa | | | | | | | | | | | | |  | |
|  |  | | |  |  | |  | | | | | | | | | | | | |  | |
| CPT-NRS |  | | |  |  | | /100 (CPT-NRS= Pain NRS on reaching CPT) | | | | | | | | | | | | |  | |
| ST_VAS 50_ |  | | |  |  | | ^0^C (ST_VAS 50_ = temp where VAS is 50) | | | | | | | | | | | | |  | |
| ST_GRD_ |  | | |  |  | | ST_GRD_= gradient of suprathreshold warm VAS curve | | | | | | | | | | | | |  | |
|  |  | | | | | |  | | | | | | | | | | | | |  | |
| Level 1 | Level 2 | | | | | | *Ethnicity Categories 2001 UK Census | | | | | | | | | | | | |  | |
| White | White British  Irish  Other White background  All white groups | | | | | |  | | | | | | | | | | | | |  | |
| Mixed | White and Black Caribbean  White and Black African  White and Asian  Other mixed background | | | | | |  | | | | | | | | | | | | |  | |
| Asian or Asian British | Indian  Pakistani  Bangladeshi  Other Asian background  All Asian groups | | | | | |  | | | | | | | | | | | | |  | |
| Black or Black British | Caribbean  African  Other Black background  All Black groups | | | | | |  | | | | | | | | | | | | |  | |
| Chinese or Other Ethnic Groups | Chinese  Other ethnic group  All Chinese or Other groups | | | | | |  | | | | | | | | | | | | |  | |
| All ethnic groups | All ethnic groups | | | | | |  | | | | | | | | | | | | |  | |
