# Supplementary material for: Sensory, psychological, and metabolic dysfunction in HIV-associated peripheral neuropathy: A cross-sectional deep profiling study
Source: Pain. 2014 Sep;155(9):1846–60. doi: 10.1016/j.pain.2014.06.014 (PMC4165602; doi:10.1016/j.pain.2014.06.014)
Supplement: Supplemental Document 2 — DFNS QST Equipment used, Heat Suprathreshold protocol and Healthy Control Inclusion and Exclusion criteria. [file mmc2.docx]

**Supplemental Document 2: *DFNS QST Equipment used, Heat Suprathreshold protocol and Healthy Control Inclusion and Exclusion criteria.***

*Thermal threshold detection*

Sensory and nociceptive thermal thresholds were determined using a SOMEDIC MSA100 thermal stimulator (SOMEDIC AB, Hörby, Sweden) using a fluid cooled Peltier element thermode measuring 25 x 50mm. Baseline temperature 32^0^C; ramped stimuli at 1^0^Cs^-1^ using method of limits. Threshold values of cold detection (CDT) and warm detection (WDT) were first determined in triplicate. A thermal sensory limen of alternating warm and cold stimuli determined the presence of paradoxical heat sensations to cold stimuli (PHS).

*Mechanical detection threshold*

MDT was assessed using a standardized set of modified von Frey filaments (Optihair2-Set, Marstock Nervtest, Schreisheim, Germany) exerting forces between 0.25 and 512 mN. The contact area of the von Frey hairs with the skin was a rounded tip (0.5 mm diameter) to avoid sharp edges that would cause nociceptor activation.

*Mechanical pain threshold*

MPT was assessed using custom-made weighted pinprick stimuli with fixed stimulus intensities (8, 16, 32, 64, 128, 256, 512 mN; flat contact area of 0.25 mm diameter; The PinPrick, MRC Systems GmbH, Heidelberg, Germany).

*Mechanical pain sensitivity and dynamic mechanical allodynia*

A stimulus-response function for MPS was determined using the same weighted pinprick stimuli as for mechanical pain threshold. Additionally, pain in response to light touch (DMA) was tested by light stroking with a cotton wisp (3 mN), a cotton wool tip fixed to an elastic strip (100 mN) and a brush (200–400 mN). Participants are requested to rate the pain of each stimulus on a numerical rating scale 0 (no pain) to 100 (most intense pain imaginable).

*Vibration detection threshold*

VDT was assessed with a Rydel–Seiffert tuning fork (128/64 Hz, 8/8 scale; Model:AB-125A, A. Barthelmes & Co. GmbH, Tuttlingen, Germany) applied at suprathreshold vibration intensity over the subjects medial malleolus until the participant indicated the sensation of vibration had ceased.

*Pressure pain threshold*

PPT was assessed by using a pressure gauge device (FDN200, Wagner Instruments, Greenwich, CT, USA) with a probe area of 1 cm^2^ that exerts pressure up to 2000 kPa.

**Suprathreshold heat protocol**

From a baseline temperature of 32^0^C, suprathreshold temperatures were reached by a ramped temperature increase of 1^0^Cs^-1^. The temperature sequence used was 46, 42, 40, 48, 50, 52, 44, 42, 48, 40, 44, 46, 50 and 52^0^C. The supratheshold temperature being tested would then be held constant for 2 sec, during which the participant was requested to rate the greatest pain experienced for the temperature being tested using a 100mm electronic visual analogue scale (eVAS) transducer (SENSEbox™ 450-010, SOMEDIC AB, Sweden). VAS anchors were defined for the participant prior to testing, and were also written on the VAS scale: from left to right on electronic VAS: 0 mm - No pain, and 100 mm - Worst pain they could imagine. Each tested temperature was separated by 30 sec. The protocol ensured that each temperature was tested twice, in a fixed, but random order, to prevent participants anticipating stimulus temperatures.

**Inclusion and Exclusion criteria for Healthy Control Subjects**

Healthy controls were volunteers 18 years and over. Inclusion of healthy volunteer data required participants to have responses to vibration, warm and cold threshold detection, within the normal standardised DFNS values. Potential participants were excluded if they presented with a neurological, vascular or psychiatric disorder or a chronic pain condition; diabetes mellitus; or were taking medication with potential central or peripheral nervous system effects, included benzodiazepines, triptans or cortisone preparations.
