# Supplementary material for: Sensory, psychological, and metabolic dysfunction in HIV-associated peripheral neuropathy: A cross-sectional deep profiling study
Source: Pain. 2014 Sep;155(9):1846–60. doi: 10.1016/j.pain.2014.06.014 (PMC4165602; doi:10.1016/j.pain.2014.06.014)
Supplement: Supplemental Document 3 — The structured neurological examination of the upper and lower limb was designed by an experienced neurologist (DB), to exam in detail, clinical neurological signs which might be present in a peripheral neuropathy. [file mmc3.doc]

The structured neurological examination of the upper and lower limb was designed by an experienced neurologist (DB), to exam in detail, clinical neurological signs which might be present in a peripheral neuropathy.

**Upper and Lower Limb Examination**

***Inspection***

The presence of any muscle wasting is noted, with special attention given to the First Dorsal Interosseous muscle in the upper limb and Extensor Digitorium Brevis in the lower limb. The presence of ulcerations and deformities is also noted.

***Motor***

Motor strength is assessed using the expanded MRC grading system (see below table for details) for shoulder abduction, elbow flexion/ extension and wrist extension in the upper limb. In the lower limb Hip flexion, Knee extension and Extensor Hallucis Longus power are assessed.

***Sensory***

Joint position proprioception – The subject’s index finger at the level of the distal-interphalangeal joint is placed in the ‘up’ and ‘down’ position, with the examiner explaining to the subject the position the joint is in when the subject’s eyes are open. The procedure is repeated with the subject’s eyes closed for 3 different joint positions. If the subject fails to recognize 3 out of 3 positions, the examiner repeats the examination on the next proximal joint and so on, until a level is reached where the subject demonstrates normal joint proprioception. This level is then recorded.

Vibration perception – the examiner applies a maximally struck 128Hz tuning fork to the distal inter-phalangeal joint of the subject’s index finger in the upper limb, and the distal interphalangeal joint in the first toe in the lower limb. The examiner asks the subject to indicate if they can feel the vibration. If they are able to detect vibration, the examiner asks if it feels the same as when applied to the subject’s sternum. If the subject indicates that it does not feel the same, the assessment is repeated at the next most proximal joint until the subject indicates that the vibration sensation felt is the same when applied distally as when applied to the sternum. This level is then recorded.

Light touch – The examiner touches the palmer aspect of fingers 1 to 5 with a cotton wisp and asks the subject to point to the finger being touched with their eyes closed. The light touch stimulus is demonstrated first to the subject on their sternum with their eyes open.

Pin-prick perception – The examiner uses a Neurotip™ pin (Owen Mumford, Oxford U.K.) asking the subject to identify if the blunt or sharp part of the Neurotip pin is being applied to the palmer aspect of the subjects index fingers. If the subject is not successful on 3 out of 3 applications, the examiner would repeat the assessment at the next most proximal joint. The level at which normal pin-prick sensation is recorded. The pin-prick examination is demonstrated first to the subject on their sternum with their eyes open

Temperature perception - The examiner uses cold and warm water filled test tubes, asking the subject to indicate if the test tube applied is warm or cold when applied to first the subjects palmer aspect of their finger tips with their eyes are closed. If the subject is unable to correctly identify the temperature, the assessment is repeated at the next most proximal joint. The examiner first demonstrates the test on the subjects sternum with their eyes open.

***Reflexes***

Using a standard 40 cm ‘Queen Square’ style reflex hammer. Reflexes assessed: biceps, triceps and brachioradialis in the upper limb; patella, Achilles’ and plantar in the lower limb. The examiner records if the reflex was normal, absent, brisk or only present with a reinforcement maneuver. Plantar reflexes are recorded as up or down.

Structured Neurological Examination CRF used in the PINS-HIV study.

| **Inspection** | **Left** | | | | | | | | | | | | | | | | | | |  | **Right** | | | | | | | | | | | | | | | | | | | | | |
| --- | --- | --- | --- | --- | --- | --- | --- | --- | --- | --- | --- | --- | --- | --- | --- | --- | --- | --- | --- | --- | --- | --- | --- | --- | --- | --- | --- | --- | --- | --- | --- | --- | --- | --- | --- | --- | --- | --- | --- | --- | --- | --- |
| 1^st^ Dorsal Interosseous | No Wasting | | | | | | | | | Wasting | | | | | | | | | |  | No Wasting | | | | | | | | | | | Wasting | | | | | | | | | | |
| Ext Digitorum Brevis | No Wasting | | | | | | | | | Wasting | | | | | | | | | |  | No Wasting | | | | | | | | | | | Wasting | | | | | | | | | | |
| Other wasting: |  | | | | | | | | |  | | | | | | | | | |  |  | | | | | | | | | | |  | | | | | | | | | | |
| Foot ulceration | Present | | | | | | | | | Absent | | | | | | | | | |  | Present | | | | | | | | | | | Absent | | | | | | | | | | |
| Foot deformity | Present | | | | | | | | | Absent | | | | | | | | | |  | Present | | | | | | | | | | | Absent | | | | | | | | | | |
| **Motor Power** |  | | | | | | | | | | | | | | | | | | | | | | | | | | | | | | | | | | | | | | | | | |
| Shoulder abduction | 5 | | 5- | | 4+ | | 4 | | 4- | | | 3 | 2 | | | 1 | | 0 |  | | 5 | 5- | | | 4+ | | | 4 | | 4- | | | 3 | | 2 | | | | 1 | | | 0 |
| Elbow flexion | 5 | | 5- | | 4+ | | 4 | | 4- | | | 3 | 2 | | | 1 | | 0 |  | | 5 | 5- | | | 4+ | | | 4 | | 4- | | | 3 | | 2 | | | | 1 | | | 0 |
| Elbow extension | 5 | | 5- | | 4+ | | 4 | | 4- | | | 3 | 2 | | | 1 | | 0 |  | | 5 | 5- | | | 4+ | | | 4 | | 4- | | | 3 | | 2 | | | | 1 | | | 0 |
| Wrist extension | 5 | | 5- | | 4+ | | 4 | | 4- | | | 3 | 2 | | | 1 | | 0 |  | | 5 | 5- | | | 4+ | | | 4 | | 4- | | | 3 | | 2 | | | | 1 | | | 0 |
| 1^st^ Dorsal Interosseous | 5 | | 5- | | 4+ | | 4 | | 4- | | | 3 | 2 | | | 1 | | 0 |  | | 5 | 5- | | | 4+ | | | 4 | | 4- | | | 3 | | 2 | | | | 1 | | | 0 |
|  | | | | | | | | | | | | | | | | | | | | | | | | | | | | | | | | | | | | | | | | | | |
| Hip flexion | 5 | | 5- | | 4+ | | 4 | | 4- | | | 3 | 2 | | | 1 | | 0 |  | | 5 | 5- | | | 4+ | | | 4 | | 4- | | | 3 | | 2 | | | | 1 | | | 0 |
| Knee extension | 5 | | 5- | | 4+ | | 4 | | 4- | | | 3 | 2 | | | 1 | | 0 |  | | 5 | 5- | | | 4+ | | | 4 | | 4- | | | 3 | | 2 | | | | 1 | | | 0 |
| Ankle dorsi-flexion | 5 | | 5- | | 4+ | | 4 | | 4- | | | 3 | 2 | | | 1 | | 0 |  | | 5 | 5- | | | 4+ | | | 4 | | 4- | | | 3 | | 2 | | | | 1 | | | 0 |
| Ext Hallucis Longus | 5 | | 5- | | 4+ | | 4 | | 4- | | | 3 | 2 | | | 1 | | 0 |  | | 5 | 5- | | | 4+ | | | 4 | | 4- | | | 3 | | 2 | | | | 1 | | | 0 |
| **Reflexes** |  | | | | | | | | | | | | | | | | | | | | | | | | | | | | | | | | | | | | | | | | | |
| Biceps | normal | | | | | absent | | | | reinforce | | | | | hyper | | | | |  | normal | | | | | absent | | | | | | reinforce | | | | | | hyper | | | | |
| Triceps | normal | | | | | absent | | | | reinforce | | | | | hyper | | | | |  | normal | | | | | absent | | | | | | reinforce | | | | | | hyper | | | | |
| Brachioradialis | normal | | | | | absent | | | | reinforce | | | | | hyper | | | | |  | normal | | | | | absent | | | | | | reinforce | | | | | | hyper | | | | |
| Patella | normal | | | | | absent | | | | reinforce | | | | | hyper | | | | |  | normal | | | | | absent | | | | | | reinforce | | | | | | hyper | | | | |
| Achilles | normal | | | | | absent | | | | reinforce | | | | | hyper | | | | |  | normal | | | | | absent | | | | | | reinforce | | | | | | hyper | | | | |
| Plantar | flexor | | | extensor | | | |  | | | | | | | | | | | |  | flexor | | | extensor | | | | |  | | | | | | | | | | | | | |
|  | | | | | | | | | | | | | | | | | | | | | | | | | | | | | | | | | | | | | | | | | | |
| **Sensory** | Upper limb | | | | | | | | | | | | | | | | | | | | | | | | | | | | | | | | | | | | | | | | | |
| Joint Position Sense norm | DIP | B of finger | | | | Mid-hand | | Wrist | | | Mid-arm | | | Elbow | | | Shoulder | | |  | DIP | | B of finger | | | | Mid-hand | | | | Wrist | | | Mid-arm | | Elbow | | | | Shoulder | | |
| Vibration Perception norm | DIP | B of finger | | | | Mid-hand | | Wrist | | | Mid-arm | | | Elbow | | | Shoulder | | |  | DIP | | B of finger | | | | Mid-hand | | | | Wrist | | | Mid-arm | | Elbow | | | | Shoulder | | |
| Light touch perception norm | DIP | B of finger | | | | Mid-hand | | Wrist | | | Mid-arm | | | Elbow | | | Shoulder | | |  | DIP | | B of finger | | | | Mid-hand | | | | Wrist | | | Mid-arm | | Elbow | | | | Shoulder | | |
| Pinprick perception norm | DIP | B of finger | | | | Mid-hand | | Wrist | | | Mid-arm | | | Elbow | | | Shoulder | | |  | DIP | | B of finger | | | | Mid-hand | | | | Wrist | | | Mid-arm | | Elbow | | | | Shoulder | | |
| Temperature perception norm | DIP | B of finger | | | | Mid-hand | | Wrist | | | Mid-arm | | | Elbow | | | Shoulder | | |  | DIP | | B of finger | | | | Mid-hand | | | | Wrist | | | Mid-arm | | Elbow | | | | Shoulder | | |
|  | Lower limb | | | | | | | | | | | | | | | | | | | | | | | | | | | | | | | | | | | | | | | | | |
| Joint Position Sense norm | DIP | B of toe | | | | Mid-foot | | M. malle | | | Lower-leg | | | Knee | | | Hip | | |  | DIP | | B of toe | | | | Mid-foot | | | | M. malle | | | Lower-leg | | | Elbow | | | | Hip | |
| Vibration Perception norm | DIP | B of toe | | | | Mid-foot | | M. malle | | | Lower-leg | | | Knee | | | Hip | | |  | DIP | | B of toe | | | | Mid-foot | | | | M. malle | | | Lower-leg | | | Elbow | | | | Hip | |
| Light touch perception norm | DIP | B of toe | | | | Mid-foot | | M. malle | | | Lower-leg | | | Knee | | | Hip | | |  | DIP | | B of toe | | | | Mid-foot | | | | M. malle | | | Lower-leg | | | Elbow | | | | Hip | |
| Pinprick perception norm | DIP | B of toe | | | | Mid-foot | | M. malle | | | Lower-leg | | | Knee | | | Hip | | |  | DIP | | B of toe | | | | Mid-foot | | | | M. malle | | | Lower-leg | | | Elbow | | | | Hip | |
| Temperature perception norm | DIP | B of toe | | | | Mid-foot | | M. malle | | | Lower-leg | | | Knee | | | Hip | | |  | DIP | | B of toe | | | | Mid-foot | | | | M. malle | | | Lower-leg | | | Elbow | | | | Hip | |

| **13 Point Expanded MRC Grading** | |
| --- | --- |
| 5 | Normal Strength |
| 5- | Uncertain muscle weakness |
| 4+ | Inability to resist maximal pressure through the full range |
| 4 | Ability to resist moderate pressure through the full range |
| 4- | Ability to resist minimal pressure through the full range |
| 3+ | Full range against gravity, resists minimal pressure through partial range |
| 3 | Full range against gravity |
| 3- | >50% range against gravity |
| 2+ | <50% range against gravity |
| 2 | Full range with gravity eliminated |
| 2- | Any arc with gravity eliminated |
| 1 | Flicker of contraction seen or palpated |
| 0 | No contraction palpable |
