# Supplementary material for: Sensory, psychological, and metabolic dysfunction in HIV-associated peripheral neuropathy: A cross-sectional deep profiling study
Source: Pain. 2014 Sep;155(9):1846–60. doi: 10.1016/j.pain.2014.06.014 (PMC4165602; doi:10.1016/j.pain.2014.06.014)
Supplement: Supplemental Document 4 — Mean values of QST parameters after z-score transformation for the three groups. Dynamic mechanical allodynia (DMA) and paradoxical heat sensations (PHS) are presented as mean number of events. ∗z-score transformed values outside 95% CI range of the DFNS normal values, percentages in brackets. CDT = Cold Detection Threshold, WDT = Warm Detection Threshold, TSL = Thermal Sensory Limen, CPT = Cold Pain Threshold, HPT = Heat Pain Threshold, MPT = Mechanical Pain Threshold, MPS = Mechanical Pain Sensitivity, WUR = Wind-Up Ratio, MDT = Mechanical Detection Threshold, VDT = Vibration Detection Threshold. [file mmc4.docx]

|  | **Healthy controls n=36** | | | | |  | **HIV-No SN n=38** | | | |  | **HIV-SN n=28** | | | | |
| --- | --- | --- | --- | --- | --- | --- | --- | --- | --- | --- | --- | --- | --- | --- | --- | --- |
|  |  |  | Number (%) of participants with: | | |  |  |  | Number (%) of participants with: | |  |  |  | | Number(%) of participants with: | |
|  | mean | SD | Loss | | Gain |  | mean | SD | Loss | Gain |  | mean | SD | | Loss | Gain |
|  |  |  | < -1.96 CI* | | >1.96 CI* |  |  |  | < -1.96 CI* | >1.96 CI* |  |  |  |  | < -1.96 CI* | >1.96 CI* |
| **CDT** | 0.02 | 0.83 | 1(2.8) | | 1 (2.8) |  | -0.68 | 0.75 | 2(5.3) | 0 |  | -1.34 | 0.93 | | 8(28.6) | 0 |
| **WDT** | -0.01 | 0.75 | 0 | | 0 |  | -0.79 | 0.69 | 1(2.6) | 0 |  | -1.31 | 0.56 | | 4(14.3) | 0 |
| **TSL** | -0.26 | 0.75 | 1(2.8) | | 0 |  | -0.86 | 0.54 | 1(2.6) | 0 |  | -1.29 | 0.67 | | 4(14.3) | 0 |
| **CPT** | -0.69 | 0.75 | 0 | | 1(2.8) |  | 0.24 | 0.71 | 0 | 0 |  | -0.18 | 0.37 | | 0 | 0 |
| **HPT** | 0.41 | 0.88 | 0 | | 2(5.6) |  | -0.59 | 1.01 | 3(7.9) | 1(2.6) |  | -1.47 | 1.05 | | 9(32.1) | 0 |
| **PPT** | -0.38 | 1.04 | 2(5.6) | | 0 |  | -1.09 | 0.87 | 7(13.2) | 0 |  | -1.29 | 1.13 | | 7(25) | 1(3.6) |
| **MPT** | 0.33 | 0.71 | 0 | | 1(2.8) |  | 0.42 | 0.88 | 0 | 1(2.6) |  | -0.01 | 0.99 | | 1(3.6) | 0 |
| **MPS** | 0.86 | 1.17 | 0 | | 7(19.4) |  | 0.36 | 1.21 | 0 | 5(13.2) |  | -0.32 | 1.06 | | 0 | 1(3.6) |
| **WUR** | -0.08 | 0.84 | 0 | | 2(5.6) |  | -0.13 | 1.12 | 1(2.6) | 2(5.3) |  | -0.21 | 1.66 | | 2(7.1) | 4(14.3) |
| **MDT** | -0.67 | 0.85 | 2(5.6) | | 0 |  | -0.77 | 0.77 | 2(5.3) | 0 |  | -2.17 | 1.06 | | 14(50) | 0 |
| **VDT** | -0.06 | 0.81 | 2(5.6) | | 0 |  | -0.84 | 1.01 | 4(10.5) | 0 |  | -2.24 | 1.62 | | 14(50) | 0 |
|  | | | | | | | | | | | | | | | | |
| **DMA** | Not present | - | - | - | |  | Not present | - | - | - |  | Not present | - | - | | - |
| **PHS events** | 0.07 | 0.24 | NA | NA | |  | 0.62 | 0.92 | NA | NA |  | 0.67 | 0.87 | NA | | NA |

**Supplemental Document 4:** Mean values of QST parameters after z-score transformation for the three groups. Dynamic mechanical allodynia (DMA) and paradoxical heat sensations (PHS) are presented as mean number of events. * z-score transformed values outside 95% CI range of the DFNS normal values, percentages in brackets. CDT= Cold Detection Threshold, WDT = Warm Detection Threshold, TSL = Thermal Sensory Limen, CPT = Cold Pain Threshold, HPT = Heat Pain Threshold, MPT = Mechanical Pain Threshold, MPS = Mechanical Pain Sensitivity, WUR = Wind-Up Ratio, MDT = Mechanical Detection Threshold, VDT = Vibration Detection Threshold.
