# Supplementary material for: Sensory, psychological, and metabolic dysfunction in HIV-associated peripheral neuropathy: A cross-sectional deep profiling study
Source: Pain. 2014 Sep;155(9):1846–60. doi: 10.1016/j.pain.2014.06.014 (PMC4165602; doi:10.1016/j.pain.2014.06.014)
Supplement: Supplemental Document 5 — Correlation of IENFD and individual thermal and mechanical QST parameters. Pearson’s correlation r values. IENFD = Intra Epidermal Fibre Density, QST = Quantitative Sensory Testing, CDT = Cold Detection Threshold, WDT = Warm Detection Threshold, TSL = Thermal Sensory Limen, CPT = Cold Pain Threshold, HPT = Heat Pain Threshold, MPT = Mechanical Pain Threshold, MPS = Mechanical Pain Sensitivity, WUR = Wind-Up Ratio, MDT = Mechanical Detection Threshold, VDT = Vibration Detection Threshold. [file mmc5.docx]

|  | **Thermal QST parameters** | | | | |  | **Mechanical QST parameters** | | | | | |
| --- | --- | --- | --- | --- | --- | --- | --- | --- | --- | --- | --- | --- |
|  | **CDT** | **WDT** | **TSL** | **CPT** | **HPT** |  | **MDT** | **MPT** | **MPS** | **WUR** | **VDT** | **PPT** |
| **r** | -0.22 | <0.01 | -0.43 | -0.21 | -0.13 |  | 0.10 | 0.00 | 0.59 | <0.01 | -0.10 | -0.05 |
| **p** | 0.29 | 0.99 | <0.05 | 0.33 | 0.55 |  | 0.63 | 0.98 | <0.05 | 0.70 | 0.67 | 0.83 |

**Supplemental Document 5**. Correlation of IENFD and individual thermal and mechanical QST parameters. Pearson’s correlation r values. IENFD = Intra Epidermal Fibre Density, QST = Quantitative Sensory Testing, CDT= Cold Detection Threshold, WDT = Warm Detection Threshold, TSL = Thermal Sensory Limen, CPT = Cold Pain Threshold, HPT = Heat Pain Threshold, MPT = Mechanical Pain Threshold, MPS = Mechanical Pain Sensitivity, WUR = Wind-Up Ratio, MDT = Mechanical Detection Threshold, VDT = Vibration Detection Threshold.
