# Supplementary material for: Sensory, psychological, and metabolic dysfunction in HIV-associated peripheral neuropathy: A cross-sectional deep profiling study
Source: Pain. 2014 Sep;155(9):1846–60. doi: 10.1016/j.pain.2014.06.014 (PMC4165602; doi:10.1016/j.pain.2014.06.014)
Supplement: Supplemental Document 6 — Mean and SD scores for NPSI pain descriptors for patients with painful HIV-SN (n = 19). NPSI = Neuropathic Pain Symptom Inventory, HIV-SN = HIV Sensory Neuropathy. [file mmc6.docx]

| NPSI measure | Painful HIV-SN |
| --- | --- |
| Burning |  |
| Mean (SD) | 4.74 (2.51) |
| Median | 5 |
| Squeezing |  |
| Mean (SD) | 3.05 (3.69) |
| Median | 1 |
| Pressure |  |
| Mean (SD) | 4.84 (2.86) |
| Median | 5 |
| Electric shock |  |
| Mean (SD) | 4.78 (3.66) |
| Median | 5 |
| Stabbing |  |
| Mean (SD) | 5.21 (3.61) |
| Median | 7 |
| Brushing evoked |  |
| Mean (SD) | 2.31 (2.75) |
| Median | 1 |
| Pressure evoked |  |
| Mean (SD) | 5.42 (3.42) |
| Median | 6 |
| Cold evoked |  |
| Mean (SD) | 3.31 (3.63) |
| Median | 1 |
| Pins and needles |  |
| Mean (SD) | 4.74 (3.74) |
| Median | 6 |
| Tingling |  |
| Mean (SD) | 6.26 (2.92) |
| Median | 7 |
| NPSI total |  |
| Mean (SD) | 44.68 (21.23) |

**Supplemental Document 6.** Mean and SD scores for NPSI pain descriptors for patients with painful HIV-SN (n=19). NPSI = Neuropathic Pain Symptom Inventory, HIV-SN = HIV Sensory Neuropathy.
