# Supplementary material for: Sensory, psychological, and metabolic dysfunction in HIV-associated peripheral neuropathy: A cross-sectional deep profiling study
Source: Pain. 2014 Sep;155(9):1846–60. doi: 10.1016/j.pain.2014.06.014 (PMC4165602; doi:10.1016/j.pain.2014.06.014)
Supplement: Supplemental Document 7 — Psychological and Sleep instruments questionnaire returned and completed per group. Numbers completed with percentages in brackets. HIV-SN = HIV sensory neuropathy, HIV–No SN = No HIV Sensory Neuropathy, ISI = Insomnia Severity Index, DAPOS = Depression Anxiety Positive Outlook Scale, PASS-20 = Pain Anxiety Symptom Scale Short Form, PCS = Pain Catastophizing Scale, SF-36 = Short Form (36) Health Survey. [file mmc7.docx]

|  | HIV-No SN n=38 | HIV-SN no pain n=7 | HIV-SN with pain n=21 |
| --- | --- | --- | --- |
|  |  |  |  |
| Questionnaire booklets returned | 32 (84.2) | 6 (85.7) | 19 (90.5) |
| ISI | 31 (81.6) | 6 (85.7) | 19 (90.5) |
| DAPOS | 29 (76.3) | 6 (85.7) | 19 (90.5) |
| PASS-20 | 29 (76.3) | 6 (85.7) | 19 (90.5) |
| PCS | 31 (81.6) | 6 (85.7) | 19 (90.5) |
| BPI | 30 (79.0) | 6 (85.7) | 19 (90.5) |
| SF-36 | 32 (84.2) | 6 (85.7) | 19 (90.5) |
| NPSI | 19 (50.0) | 4 (57.1) | 19 (90.5) |

**Supplemental Document 7.** Psychological and Sleep instruments questionnaire returned and completed per group. Numbers completed with percentages in brackets. HIV-SN = HIV sensory neuropathy, HIV-No SN = No HIV Sensory Neuropathy, ISI = Insomnia Severity Index, DAPOS = Depression Anxiety Positive Outlook Scale, PASS-20 = Pain Anxiety Symptom Scale Short Form, PCS = Pain Catastophizing Scale, SF-36 = Short Form (36) Health Survey.
